# Proteome-Wide Identification and Functional Analysis of Lysine Crotonylation in *Trichophyton rubrum* Conidial and Mycelial Stages

**DOI:** 10.3389/fgene.2022.832668

**Published:** 2022-03-10

**Authors:** Xingye Xu, Xiangqi Hu, Jie Dong, Ying Xue, Tao Liu, Qi Jin

**Affiliations:** NHC Key Laboratory of Systems Biology of Pathogens, Institute of Pathogen Biology, Chinese Academy of Medical Sciences and Peking Union Medical College, Beijing, China

**Keywords:** post-translational modification, crotonylation, proteome, fungus, *Trichophyton rubrum*

## Abstract

Lysine crotonylation is a newly discovered post-translational modification (PTM) with key roles in various important regulatory pathways. Despite its functional significance, there is limited knowledge about crotonylation in fungi. *Trichophyton rubrum* is the most common fungal pathogen in human infection and is considered a model organism of dermatophytes and human pathogenic filamentous fungi. In this study, we obtained a proteome-wide crotonylation profile of *T. rubrum*, leading to the identification of 14,019 crotonylated sites on 3144 proteins. The crotonylated proteins were significantly involved in translation and in various metabolic and biosynthetic processes. Some proteins related to fungal pathogenicity were also found to be targets of crotonylation. In addition, extensive crotonylation was found on histones, suggesting a role in epigenetic regulation. Furthermore, about half of the crotonylated proteins were specific to either the conidial or the mycelial stage, and functional enrichment analysis showed some differences between the two stages. The results suggest that the difference in crotonylation between the two stages is not due to differences in protein abundance. Crosstalk of crotonylation with acetylation, propionylation, and succinylation suggests distinct regulatory roles. This study is the first crotonylation analysis in dermatophytes and human pathogenic filamentous fungi. These results represent a solid foundation for further research on PTM regulatory mechanisms in fungi and should facilitate improved antifungal strategies against these medical important species.

## Introduction

Dermatophytes, which mainly cause superficial mycosis, are among the most prevalent human infectious pathogens worldwide (Fekrazad et al., 2017). *Trichophyton rubrum* is responsible for the majority of dermatophyte infections (Ghannoum and Isham, 2014; Xiao et al., 2019). This fungus specifically invades keratinized tissues such as hair, nails, and cutaneous stratum corneum (Huang et al., 2015). In addition, *T. rubrum* is capable of deep invasion of internal organs such as the lymph nodes, bones, and brain, especially in immunocompromised patients (Zheng et al., 2020; Wang et al., 2021). This infection presents an important public health concern owing to persistent prevalence, frequent relapse, and an increase in invasive infections (Xu et al., 2015; Bitencourt et al., 2016). Limited antifungal drugs are available for clinical use and those that are commonly used cause many side effects including hepatotoxicity (Wingard et al., 1999; Robles-Martinez et al., 2019; Luo et al., 2021). Moreover, *T. rubrum* has been reported to show resistance to some antifungal drugs in several cases (Monod, 2019; Monod et al., 2019). Deeper understanding of the features of *T. rubrum* may provide a basis to develop improved strategies for combating this medically important fungus. However, limited genetic information is available for this fungus, in contrast to others such as *Candida albicans* and *Aspergillus* spp. (Alshahni and Yamada, 2017).

*T. rubrum* has been studied as a model organism of dermatophytes and anthropophilic pathogenic filamentous fungi (Liu et al., 2007). It has two major growth stages: the dormant conidia, which can persist in adverse environments, and the mycelia, which represent a longitudinal growth stage that aggravates infection after conidia adhere to the host (Liu et al., 2007). Some studies have revealed the biological characteristics of this fungus at the genomic, transcriptomic, and proteomic levels (Persinoti et al., 2014; Bitencourt et al., 2016; Wang et al., 2018; Xu et al., 2018; Zheng et al., 2020). Post-translational modifications (PTMs) are also important biological features of this fungus. First, PTMs on histones represent an essential epigenetic mechanism that regulates transcription, DNA replication, and DNA repair (Bannister and Kouzarides, 2011). These histone PTMs are dynamically added or removed in response to different states, and aberrant histone PTMs are often related to various diseases (Gong and Miller, 2019; Kollenstart et al., 2019). Second, PTMs are also found on non-histone proteins (Liu et al., 2014), where they provide an elegant regulatory mechanism to control protein function; this is much more efficient than regulation of protein turnover (Narita et al., 2019). Different PTMs on a single protein can generate distinct isoforms with varying functions that greatly increase the functional diversity of the proteome (Aebersold et al., 2018). Hundreds of different types of PTMs are considered to exist in eukaryotic organisms, including phosphorylation, acetylation, methylation, ubiquitylation, glycosylation, and succinylation (Haodong Xu et al., 2017; Narita et al., 2019). Multiple PTMs can positively or negatively affect each other, termed PTM crosstalk, and work in concert to regulate protein function (Horita et al., 2018; Wu et al., 2019). In our previous studies, three types of PTM were identified in *T. rubrum*, including lysine acetylation, propionylation, and succinylation (Xingye Xu et al., 2017; Xu et al., 2018; Xu et al., 2019). All these PTMs show distinct characteristics, and they are all involved in various essential pathways.

Lysine crotonylation is a newly discovered PTM that was first identified on histones by Tan et al. (Tan et al., 2011). Subsequently, many non-histone proteins were identified to be crotonylated, including some thought to be involved in many key pathways (Weizhi Xu et al., 2017; Wei et al., 2017; Wu et al., 2017; Yang et al., 2018). For instance, in the small intestine crypt and colon, crotonylation on histone H3 was found to be most abundant at the lysine 18 site (H3K18cr), suggesting that increased H3K18cr abundance at transcription start sites was associated with higher gene expression levels (Fellows et al., 2018). In addition, crotonylation is involved in cancers. Crotonylation levels have been reported to be increased in the thyroid, esophagus, colon, pancreas, and lung carcinomas and decreased in the liver, stomach, and kidney carcinomas (Wan et al., 2019). Furthermore, crotonylation levels were shown to be correlated with the TNM (tumor, node, and metastasis) stage in hepatocellular carcinoma (Wan et al., 2019). Regarding fungal species, a large-scale lysine crotonylome analysis performed in *C. albicans* revealed that crotonylation was mainly involved in biosynthetic events and carbon metabolism (Zhou et al., 2021). However, knowledge of crotonylation in fungi is still insufficient; in particular, no study of crotonylation has been performed in dermatophytes or human pathogenic filamentous fungi.

In this study, we performed a proteome-wide identification of lysine crotonylation in *T. rubrum* and compared the conidial and mycelial stages. We also analyzed the functions and roles of crotonylated proteins and the amino acid motifs of crotonylated sites, as well as the crosstalk of crotonylation with acetylation, propionylation, and succinylation. These results form a foundation for future research on the PTM regulatory mechanism in *T. rubrum* and closely related species and provide new insights regarding these significant pathogenic fungi.

## Materials and Methods

### Strain Culture and Collection

The *T. rubrum* strain BMU01672 was incubated on potato dextrose agar (Becton Dickinson, Sparks, MD, United States) at 28°C for 3 weeks to induce conidia. Approximately, 100 µL of the conidia dilution (5 × 10^4^ CFU/ml) was inoculated in 100 ml Sabouraud liquid medium (Becton Dickinson) and incubated at 28°C with constant shaking to culture mycelia. Conidia were collected with ice-cold distilled water and then filtered through Miracloth (Merck, Billerica, MA, United States). Mycelia were thoroughly washed with distilled water and collected through centrifugation.

### Protein Extraction and Digestion

The conidia and mycelia samples were ground in liquid nitrogen; then, the cell powder was sonicated on ice three times in lysis buffer (8 M urea, 10 mM dithiothreitol (DTT), 3 µM trichostatin A (TSA), 50 mM nicotinamide (NAM), and 1% protease inhibitor cocktail). The supernatant was collected, and the protein was precipitated with cold 15% trichloroacetic acid (TCA) at −20°C overnight. The protein precipitate was washed with cold acetone three times. Proteins were redissolved in 8 M urea (100 mM NH_4_HCO_3,_ pH 8.0), and the protein concentration was determined with a Pierce BCA Protein Assay Kit (Thermo Scientific, Rockford, IL, United States).

For digestion, proteins were reduced with 10 mM DTT and alkylated with 20 mM IAA. Then, the protein solution was diluted to give a urea concentration of less than 1 M. Finally, trypsin (Promega, Madison, WI, United States) was added at a trypsin/protein ratio of 1:50 (w/w), followed by incubation overnight.

### High-Performance Liquid Chromatography Fractionation and Enrichment of Crotonylated Peptides

Peptide samples were fractionated by high-pH reverse-phase HPLC using an Agilent 300 Extend C18 column (5 μm particles, 4.6 mm ID, 250 mm length; Agilent Technologies, Santa Clara, CA, United States) into 80 fractions. Then, the peptides were combined into six fractions per sample according to a previously described method (Xu et al., 2018). All the combined fractions were dried by vacuum centrifugation.

The peptides were dissolved in the NETN buffer solution (1 mM EDTA, 50 mM Tris-HCl, 100 mM NaCl, 0.5% NP-40, pH 8.0), and the supernatant was incubated with pre-conjugated anti-crotonylated lysine (Kcr) agarose antibody beads (PTM Biolabs, Hangzhou, China) with gentle shaking at 4°C overnight. To remove nonspecifically bound peptides, the resin beads were washed four times with the NETN buffer and twice with deionized water. The peptides remaining bound to the resin were eluted using 0.1% trifluoroacetic acid. Finally, the peptides were cleaned with C18 ZipTips (Millipore, Billerica, MA, United States) and redissolved in 0.1% formic acid for subsequent analysis.

### Liquid Chromatography–Tandem Mass Spectrometry Identification of Crotonylated Peptides

The peptides were loaded onto a reverse-phase pre-column (Acclaim PepMap 100, Thermo Scientific, Waltham, MA, United States) and separated using a reverse-phase analytical column (Acclaim PepMap RSLC, Thermo Scientific). The gradient was as follows: solvent B (0.1% formic acid in 98% acetonitrile) increasing from 7 to 25% for 24 min, 25–40% for 8 min, climbing to 80% in 5 min, and held at 80% for 3 min, with a constant flow rate of 400 nL/min on an EASY-nLC 1000 system (Thermo Scientific).

The eluted peptides were directly subjected to MS/MS and analyzed online with Q Exactive™ Plus (Thermo Scientific). The primary peptides were detected at a resolution of 70,000, and the m/z scan range was 350–1800. The ion count threshold was 1 E^4^ in the MS survey scan with 10 s dynamic exclusion. Fragmented ions were generated using higher-energy collisional dissociation (HCD) at 28% normalization collision energy (NCE), and the secondary scan was detected at a resolution of 17,500. A data-dependent acquisition (DDA) mode was adopted, with one MS scan followed by 20 MS/MS scans. Automatic gain control (AGC) was used, and 5E^4^ ions were accumulated for generation of MS/MS spectra. Three replicates were performed for each stage (conidial and mycelial).

The *T. rubrum* protein database was downloaded from https://www.ncbi.nlm.nih.gov/bioproject/38221 (including 10,418 protein sequences and commonly observed contaminants added to the database). The obtained MS/MS data were searched against the *T. rubrum* protein database concatenated with a reverse decoy database, using MaxQuant with its integrated Andromeda search engine (v.1.5.2.8). The following parameters were set for protein and peptide identification: trypsin/P was specified as the cleavage enzyme and allowed up to four missing cleavages; at most, five modifications per peptide were allowed; a mass error of 10 ppm was set for precursor ions and 0.02 Da for fragment ions; carbamidomethylation on Cys was specified as fixed modification; and crotonylation on Lysine, oxidation on Met, and acetylation on the protein N-terminal were specified as variable modifications. The minimum peptide length was set to 7. A false discovery rate (FDR) < 1% was set for proteins, peptides, and modification sites. The score for peptide identification was set to >40. The probability of site localization was set to > 0.75.

### Peptide Synthesis and Parallel Reaction Monitoring Analysis

Two crotonylated peptides, SDK(cr)ETAPVQATEQHDEK and SK(cr)IDELKPEFEDK, were synthesized (Chinese Peptide Company, Hangzhou, Zhejiang, China) and then separated and identified with LC-MS/MS. Synthetic peptide samples were eluted on a UPLC system with identical flow rates and gradient settings for crotonylated peptide identification in the DDA mode. All the eluted peptides were measured online using the Q Exactive™ Plus (Thermo Scientific) apparatus in the data-independent acquisition PRM mode. The full mass spectra covered the m/z range of 350–1000, and peptides were detected at a resolution of 70,000. The MS/MS scans targeted the specific peptides in the inclusion list (Supplementary Table S1). The peptides were fragmented with HCD at a 28% NCE and scanned at a resolution of 17,500. The AGC target was 5 E^4^, and the maximum injection time was 200 ms.

A spectral library was created in Skyline by importing the search results from the DDA identification described previously. Data obtained from the PRM assay were matched to specific peptides in the spectral library. The parameters were set as follows: 1) enzyme: trypsin (KR/P); 2) fixed modification: carbamidomethylation on Cys; 3) dynamic modifications: oxidation on Met and crotonylation on Lys; 4) max missed cleavage: 0; and 5) peptide length: 7–25. Transition settings: 1) precursor charges: 2 and 3; 2) ion charges: 1; 3) ion types: b and y; 4) product ion selection: from ion 3 to the last ion; and 5) ion match tolerance: 0.02 Da. The ‘dotp’ value represents a dot-product similarity metric between the PRM peak areas and the MS/MS library peak intensities. A value closer to 1.0 indicates a better match.

### Western Blot Analysis

Twenty micrograms of each of the whole-cell protein samples for conidia and mycelia were separated by 12% sodium dodecyl polyacrylamide gel electrophoresis and then transferred to polyvinylidene fluoride membranes (Thermo Scientific). The membranes were blocked in 3% bovine serum albumin for 1 h and subsequently incubated with the anti-histone H3K14cr antibody and anti-histone H3 antibody (PTM Biolabs) at 4°C overnight. After washing with Tris-buffered saline with Tween 20 three times, the membranes were incubated with the horseradish-peroxidase-linked anti-rabbit secondary antibody. Finally, the bands were detected with SuperSignal West Pico PLUS Chemiluminescent Substrate (Thermo Scientific).

### Bioinformatics Analysis

Gene ontology (GO) annotations were derived from the UniProt-GOA database (http://www.ebi.ac.uk/GOA/). The identified protein ID was converted to UniProt ID and then mapped to GO ID. For proteins that were not annotated by the UniProt-GOA database, the GO function of proteins was annotated based on the protein sequence alignment by InterProScan software (version 5) (Jones et al., 2014). The annotated proteins were classified at the level two GO annotation based on three categories: the biological process, molecular function, and cellular component.

GO enrichment analysis was performed at all the GO levels. A two-tailed Fisher’s exact test was employed for enrichment of the identified proteins against all proteins in the database. Correction for multiple hypothesis testing was performed using a standard FDR control method. A corrected *p*-value < 0.05 was considered significant.

The pathways involving crotonylated proteins were annotated based on the Kyoto Encyclopedia of Genes and Genomes (KEGG) database using KEGG online service tools KAAS (Moriya et al., 2007). The annotated proteins were mapped to the pathways using the KEGG Mapper. The cutoff value of *p* < 0.05 was applied in the KEGG enrichment analysis.

Subcellular localization of crotonylated proteins was predicted with WoLF PSORT (version II) (Horton et al., 2007). The organism type was specified as fungi. The proteins were classified into basic cellular components of the eukaryotic cell.

We used motifs-x software to analyze the sequence patterns surrounding the Kcr (Chou and Schwartz, 2011). The heat map of motif analysis was generated using the pheatmap package (version 0.6.1) of R language with default parameters.

## Results

### Identification of Crotonylated Proteins and Sites

Based on the enrichment of crotonylated peptides and high-throughput LC-MS/MS analysis, we performed a proteome-wide identification of the crotonylated sites in *T. rubrum*. The mass errors for all the identified peptides were near zero, and most of them were <10 ppm. The scores for peptide identification were all above 40 (Supplementary Figure S1). In addition, the lengths of most peptides were between 8 and 20 amino acids, consistent with the properties of peptides digested by trypsin (Supplementary Figure S2). All these results suggest the high quality and reliability of our proteome data.

**FIGURE 1:**
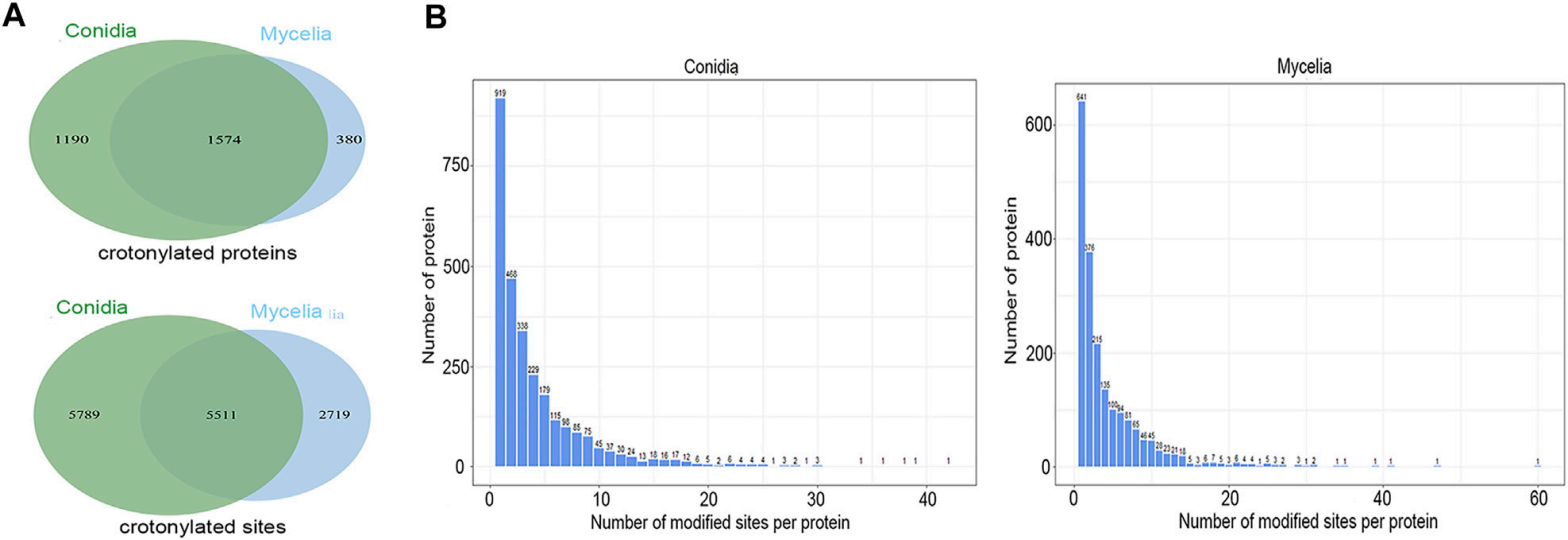
Crotonylation modifications identified in *T. rubrum*. **(A)** Crotonylated proteins and sites identified in the conidial and mycelial stages, respectively. **(B)** Numbers of crotonylated sites per protein in the conidial and mycelial stages, respectively.

In total, 14,019 crotonylated sites on 3144 proteins were identified, accounting for 30% of the *T. rubrum* proteome. Among these, 11,300 crotonylated sites on 2764 proteins were identified in the conidial stage, and 8230 crotonylated sites on 1954 proteins were identified in the mycelial stage (Supplementary Table S2). When compared between the two stages, 1574 proteins were modified both in the conidial and mycelial stages, 1190 crotonylated proteins were specific to conidia, and 380 crotonylated proteins were specific to mycelia. In addition, 5511 crotonylated sites were common to the two stages, 5789 crotonylated sites were specific to conidia, and 2719 crotonylated sites were specific to mycelia (Figure 1A). Furthermore, crotonylation modification had a high site-per-protein ratio; the average site-per-protein ratio was 4.09 in conidia and 4.21 in mycelia (Figure 1B). The protein TERG_00084T3 was the most heavily crotonylated protein, with 60 crotonylated sites in mycelia and 17 crotonylated sites in conidia.

### Validation of Crotonylated Site Identification by the PRM Assay of Synthesized Peptides

**FIGURE 2:**
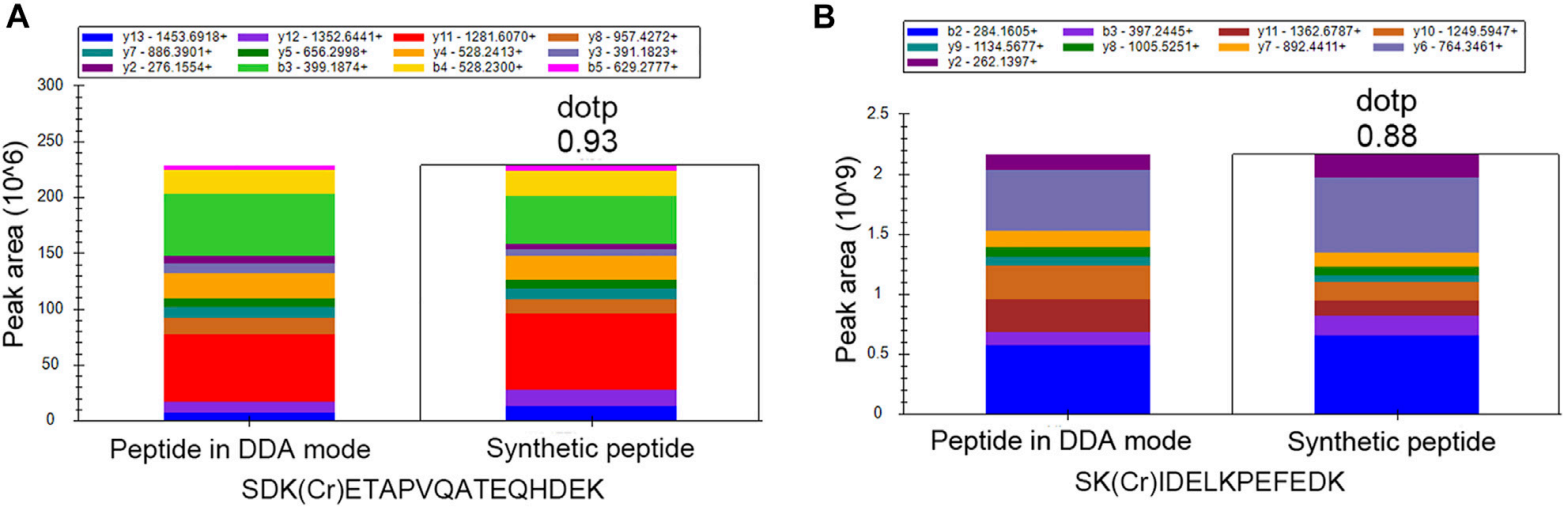
PRM validation of two crotonylated peptides. Comparison between synthetic peptides identified in the PRM mode and the corresponding peptides identified in the DDA mode for **(A)** SDK(Cr)ETAPVQATEQHDEK and **(B)** SK(Cr)IDELKPEFEDK.

In order to further validate the crotonylation modifications identified in our study, we synthesized two crotonylated peptides in accordance with two randomly selected crotonylated peptides identified in the DDA mode in our study. These two synthetic peptides were separated by liquid chromatography with identical gradients to those used for their counterparts and then identified by MS/MS in the PRM mode. Owing to their specific amino acid composition and modification, each peptide had specific retention time and MS/MS spectra. The retention times of the synthetic peptides and their counterparts are shown in Supplementary Figure S3, showing high consistency between the two pairs of counterparts. In addition, the MS/MS spectra of the two synthetic peptides identified in the PRM mode were compared with the spectra of their counterparts identified in the DDA mode. As shown in Figure 2, the similarity of the spectra for each synthetic peptide and its counterpart was evaluated based on the “dotp” value, where a value closer to 1.0 indicates a better match. The two peptides SDK(Cr)ETAPVQATEQHDEK and SK(Cr)IDELKPEFEDK showed “dotp” values of 0.93 and 0.88, respectively, suggesting high similarities. All these results suggest the reliability of our crotonylome data.

### Functional Classification and Subcellular Location Analysis of the Crotonylated Proteins

**FIGURE 3:**
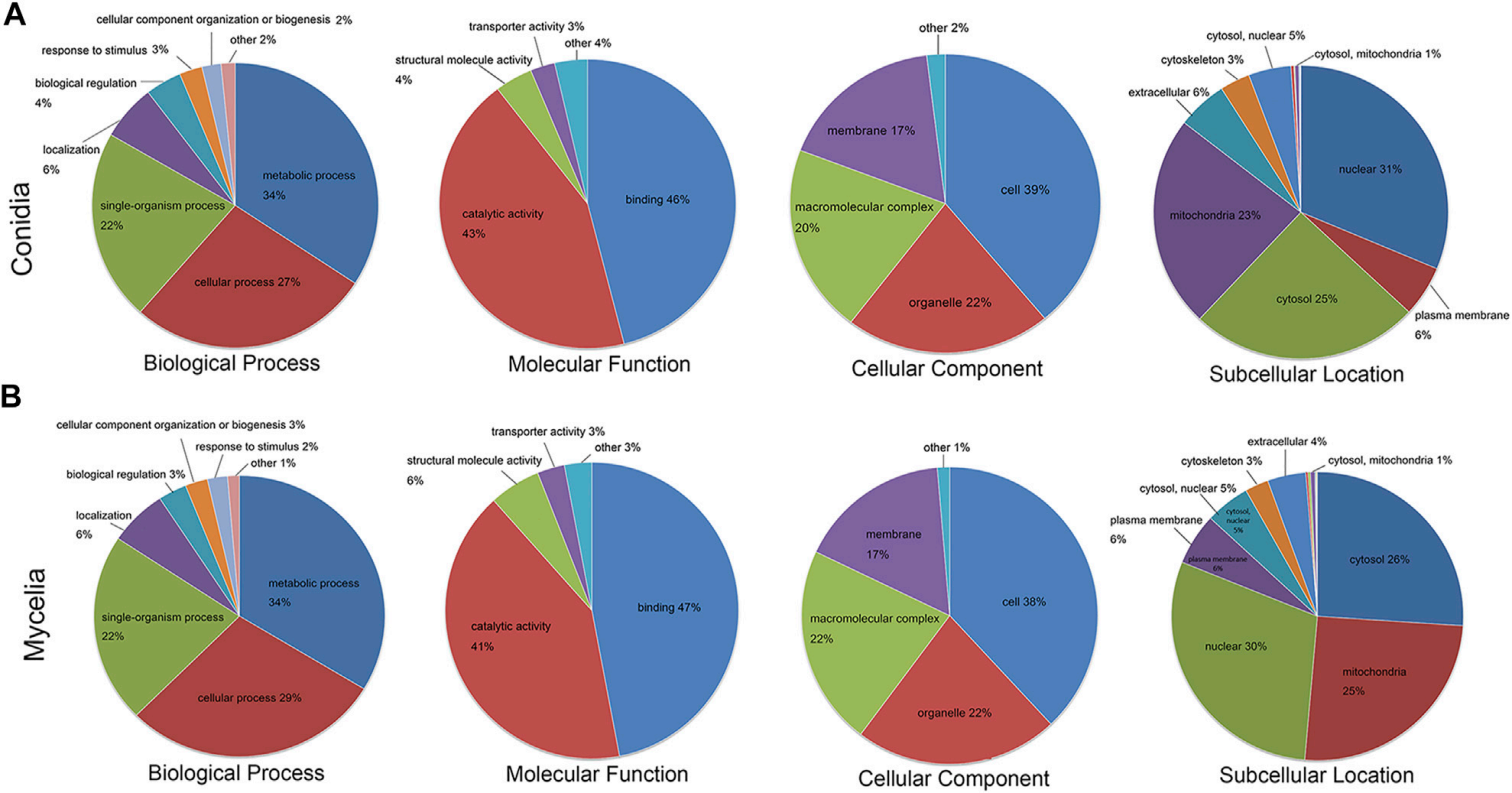
Functional annotation of the crotonylated proteins. GO biological process, molecular function and cellular component classifications, and subcellular localization analysis of the crotonylated proteins. The crotonylated proteins in the **(A)** conidial stage and **(B)** mycelial stage were subjected to differentiate function classification.

The crotonylated proteins in the conidial and mycelial stages were classified based on GO terms and subcellular location. As shown in Figure 3 and Supplementary Tables S3, S4, the crotonylated proteins showed similar functional distributions in conidia and mycelia. In the biological process classification, the metabolic process was the largest category for both the conidial and mycelial stages (34% in both conidia and mycelia), followed by the cellular process (27% in conidia and 29% in mycelia) and single-organism process (22% in both conidia and mycelia). In the molecular function classification, binding (46% in conidia and 47% in mycelia) and catalytic activity (43% in conidia and 41% in mycelia) were the two major categories. According to the cellular component classification, the crotonylated proteins were involved in four major categories: the cell (39% in conidia and 38% in mycelia), organelle (22% in both conidia and mycelia), macromolecular complex (20% in conidia and 22% in mycelia), and membrane (17% in both conidia and mycelia). In the subcellular location classification, the nucleus was the largest component (31% in conidia and 30% in mycelia), followed by cytosol (25% in conidia and 26% in mycelia) and mitochondria (23% in conidia and 25% in mycelia). Some crotonylated proteins were also predicted to be located in the plasma membrane, cytoskeleton, and Golgi apparatus.

### Functional Enrichment Analysis of Crotonylated Proteins

**FIGURE 4:**
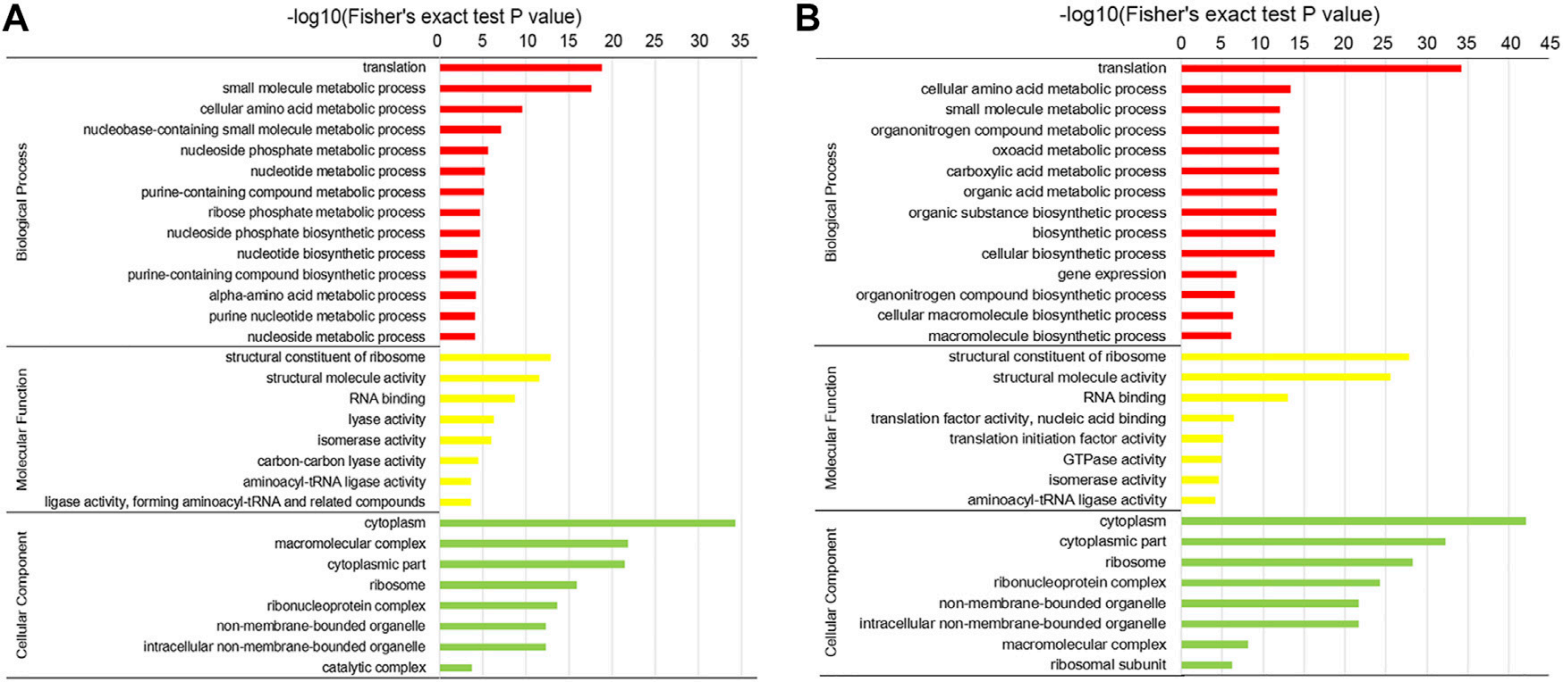
GO enrichment of the crotonylated proteins. Enrichment analysis of crotonylated proteins in the **(A)** conidial stage and **(B)** mycelial stage.

The crotonylated proteins were subjected to GO and KEGG enrichment analyses to illustrate their functional significance. As shown in Figure 4 and Supplementary Table S5, according to the GO enrichment analysis, with respect to biological processes, translation, small molecule metabolic process, and cellular amino acid metabolic process were the three most significantly enriched items in both the conidial and mycelial stages. In addition, various metabolic processes and biosynthetic processes were significantly enriched, including the nucleotide metabolic process, ribose phosphate metabolic process, and nucleotide biosynthetic process in conidia and the oxoacid metabolic process, biosynthetic process, and macromolecule biosynthetic process in mycelia. In the molecular function class, the structural constituent of ribosomes, structural molecule activity, RNA binding, isomerase activity, and aminoacyl-tRNA ligase activity were significantly enriched in both the stages. Lyase activity, carbon–carbon lyase activity, and ligase activity, forming aminoacyl-tRNA and related compounds, were enriched in conidia; and the translation factor activity, nucleic acid binding, translation initiation factor activity, and GTPase activity were enriched in mycelia. Most terms enriched in the molecular function class were related to ribosome- and translation-related roles. In the cellular component class, the ribosome, ribonucleoprotein complex, and ribosomal subunit were significantly enriched, supporting the results for the biological process and molecular function classes that indicated that crotonylated proteins were involved in translation.

**FIGURE 5:**
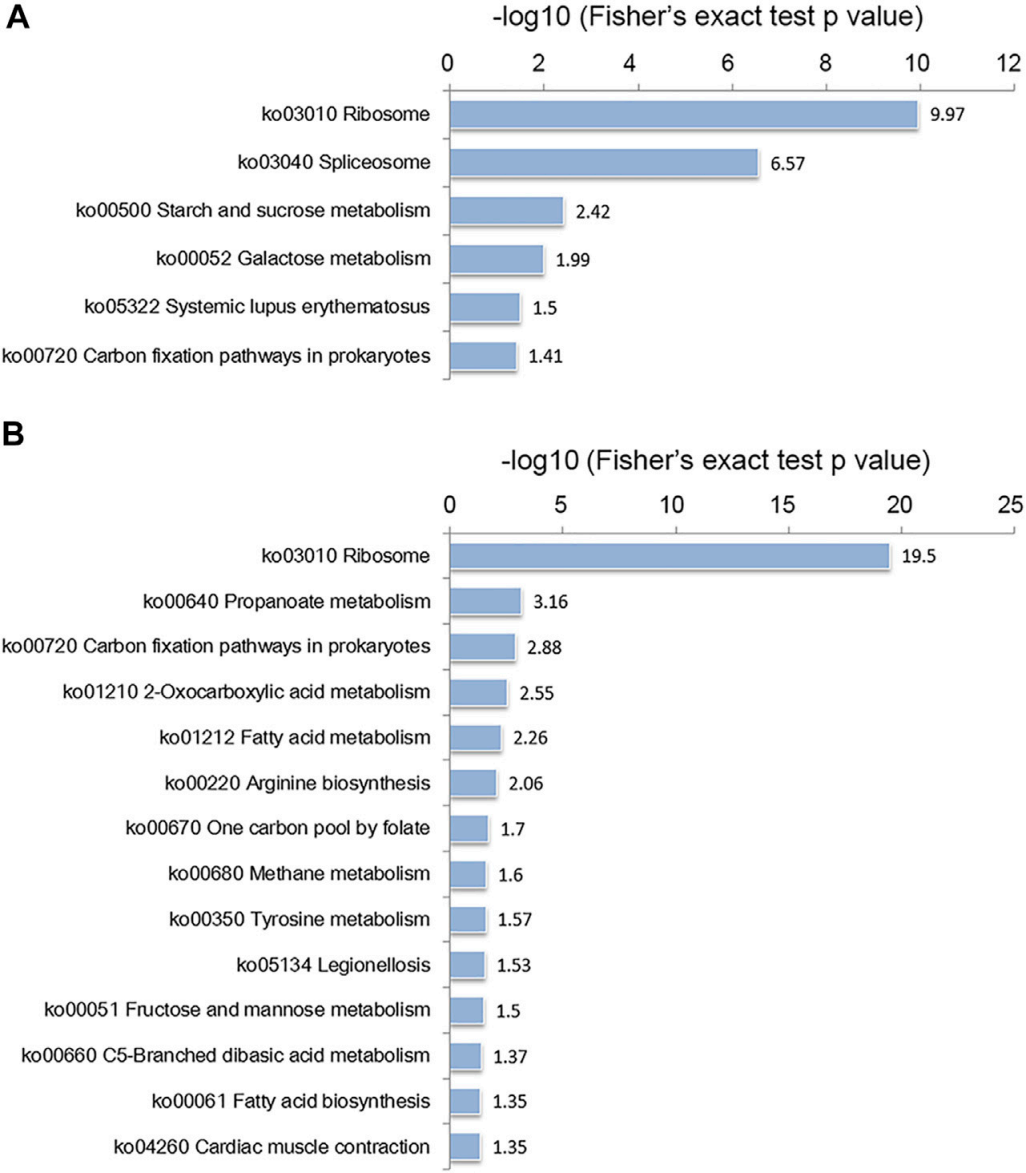
KEGG enrichment analysis of crotonylated proteins in the **(A)** conidial stage and **(B)** mycelial stage.

According to the KEGG enrichment analysis, as shown in Figure 5, the ribosome was the most significantly enriched item in both the conidial and mycelial stages, consistent with the results of the GO enrichment analysis. As shown in Supplementary Figure S4 and Supplementary Table S6, 92 ribosomal proteins were modified by crotonylation. In addition, these ribosomal proteins were extensively crotonylated, with an average ratio of modified sites per ribosomal protein of 6.8. L7Ae was the most heavily modified ribosomal protein, with 21 crotonylated sites, followed by L3e (19 crotonylated sites) and S4e (15 crotonylated sites). Most of these ribosomal proteins were modified in both stages, except for L11 and L29e, which were specifically modified in the conidial stage, and L13, S9, S2, S16, and S26e, which were specifically modified in the mycelial stage. When the modified sites of ribosomal proteins were compared between the two stages, the mycelial stage showed more abundant crotonylation than the conidial stage; a total of 590 crotonylated sites on 87 ribosomal proteins were identified in mycelia, whereas 483 crotonylated sites on 90 ribosomal proteins were identified in conidia. In addition, some KEGG pathways were specifically enriched in the conidial or mycelial stage: spliceosome, starch and sucrose metabolism, galactose metabolism, and systemic lupus erythematosus were specifically enriched in conidia, whereas propanoate metabolism, fatty acid metabolism, methane metabolism, and fatty acid biosynthesis were specifically enriched in mycelia (Figure 5 and Supplementary Table S7).

### Crotonylated Proteins Related to Fungal Pathogenicity

Among the identified crotonylated proteins, some were considered to be related to fungal virulence and pathogenicity. Dermatophytes are almost exclusively localized in keratinous tissues (Sriranganadane et al., 2011; Mercer and Stewart, 2019). Thus, the keratinolytic enzymes, including secreted proteases and peptidases, have key roles in infection of keratinous tissues and are considered to be important virulence factors in dermatophytes (Nenoff et al., 2014). In addition, antifungal resistance posed a severe problem in the treatment of fungal infections (Costa et al., 2014). Efflux transporters can extrude drugs from the cytosol and are major contributors to drug resistance (Garbinski et al., 2019). The currently known efflux superfamilies in fungi comprise two main types: the ATP-binding cassette (ABC) and major facilitator superfamily (MFS) transporters. These two types of efflux transporters are also considered to be related to fungal pathogenicity (Perez-Cantero et al., 2020). In our study, 45 proteases or peptidases and 30 efflux transporters were identified to be crotonylated (Supplementary Table S8). Some of these proteins were extensively modified by crotonylation. For instance, aminopeptidase (TERG_06767T0) has 37 crotonylated sites, metallopeptidase MepB (TERG_05923T0) has 23 crotonylated sites, and the ABC transporter (TERG_03988T0) has 16 crotonylated sites.

### Crotonylation on Histones

**FIGURE 6:**
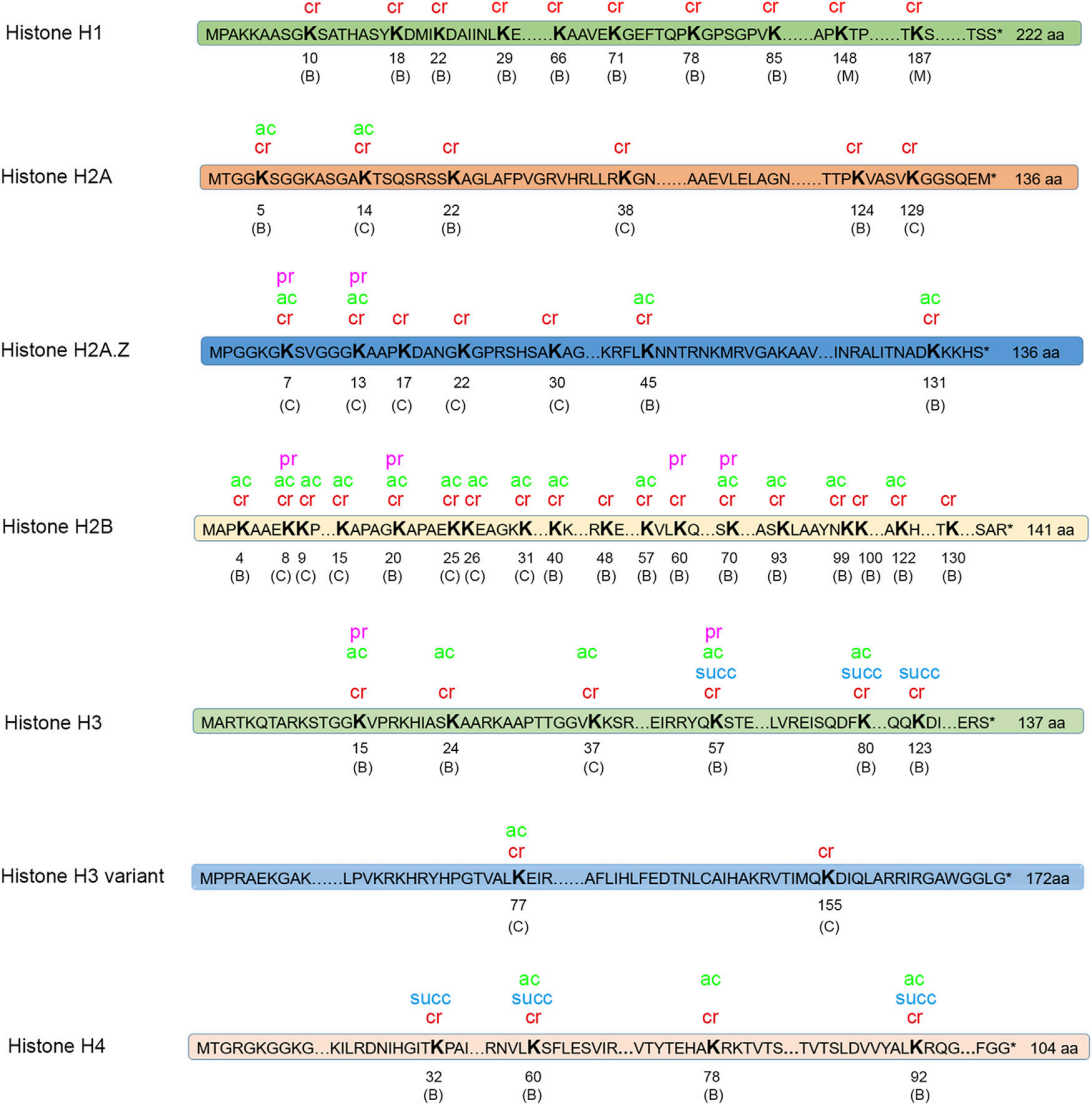
Modifications on histones. Four types of modification were identified on histones. Cr, crotonylation; ac, acetylation; pr, propionylation; succ, succinylation. “C” in parentheses indicates the site is only crotonylated in conidia; “M” in parentheses indicates that the site is only crotonylated in mycelia; “B” in parentheses indicates that the site is crotonylated in both conidia and mycelia.

PTM of histones is an important mechanism of epigenetic regulation. Dynamic alteration of PTMs on histones has been illustrated to affect gene transcription (Kurdistani and Grunstein, 2003; Li et al., 2007). In our study, a total of 53 crotonylated sites were identified on histones, as shown in Figure 6. Among these crotonylated sites, 34 sites were modified in both the conidial and mycelial stages. In addition, 17 sites were specific to the conidial stage, and two sites were specific to the mycelial stage. For instance, the histone site H3K14 was commonly crotonylated in both stages. Western blot analysis was performed using a site-specific crotonylated antibody anti-H3K14cr to validate crotonylations at this site. As shown in Supplementary Figure S5, crotonylation modification was detected at this site in both stages, consistent with the LC-MS/MS results.

Histones are also targets for other types of PTMs. The crotonylated sites on histones were compared with acetylated, succinylated, and propionylated sites on histones identified in *T. rubrum* in our previous studies. In total, 32 crotonylated histone sites were also found to be modified by at least one of the other three types of PTM. Notably, one histone site H3K56 (amino acid position calculation is started from the amino acid next to the initial M) was modified by all four types of PTM. H2B was the most extensively modified histone by crotonylation (18 crotonylated sites) and was also significantly modified by acetylation (14 sites) and propionylation (four sites). However, the functional significance of the extensively modified histone site H3K56 and histone H2B needs further exploration.

### Motif Analysis Surrounding the Crotonylated Sites

The sequences surrounding the Kcr were analyzed using motif-x, which analyzes the amino acids in specific positions (10 amino acids upstream and downstream of the Kcr). As shown in Supplementary Figure S6, 19 and 14 significantly enriched and conserved crotonylated site motifs were identified in the conidial and mycelial stages, respectively. Among these, 10 motifs were common to the two stages: EKcr, KcrE, KcrD, Kcr**E, Kcr**D, K******Kcr, DKcr, YKcr, AKcr, and K*******Kcr. In addition, nine motifs (KcrE***K, GKcr, Kcr***E, Kcr*E, Kcr*D, FKcr, NKcr, D*Kcr, and Kcr***D) were specifically enriched in the conidial stage, and four motifs (K*****Kcr, E*Kcr, KcrV, and K*********Kcr) were specifically enriched in the mycelial stage.

**FIGURE 7:**
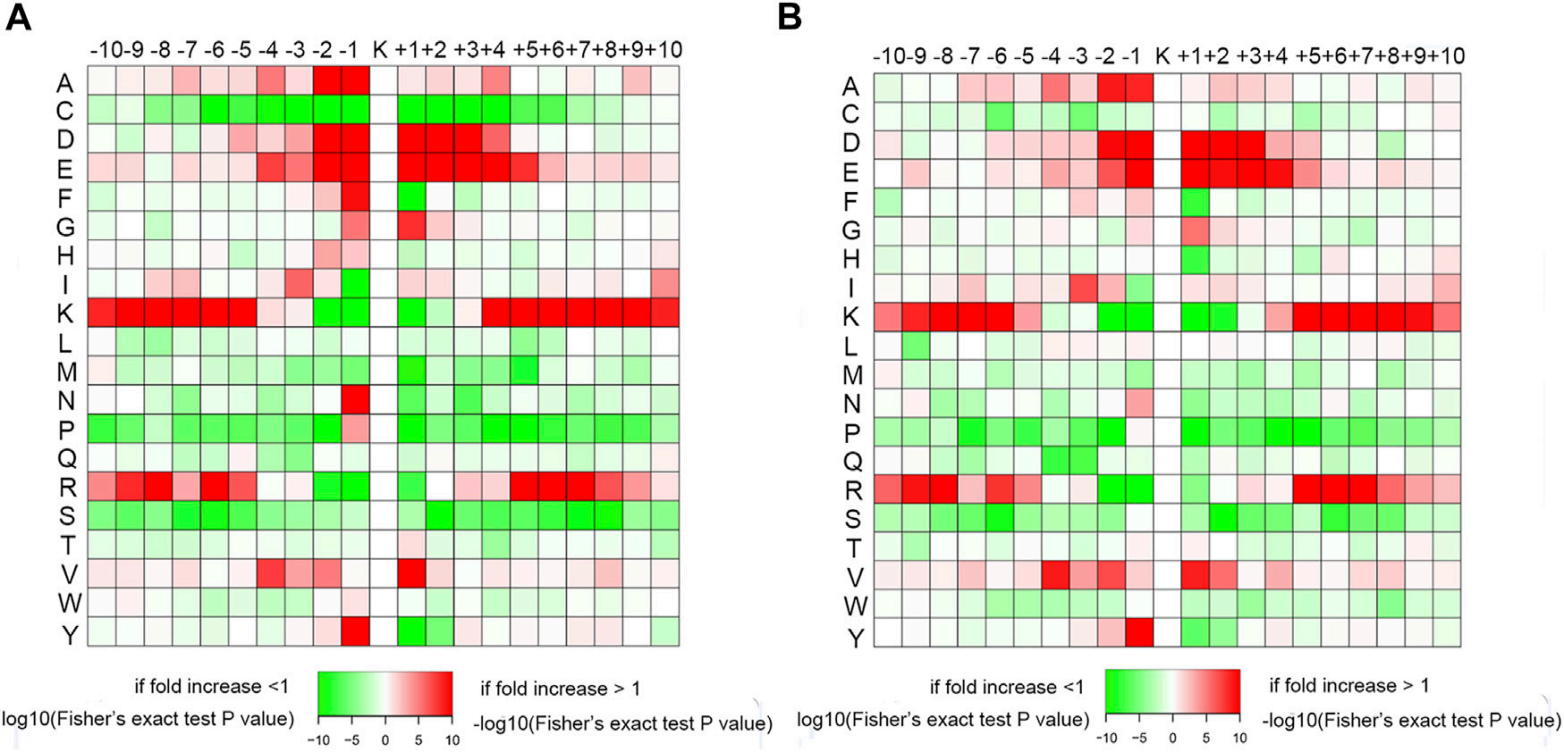
Heat maps of amino acid sequences around the Kcr residues in the **(A)** conidial stage and **(B)** mycelial stage. The red color represents enrichment, and green represents depletion of amino acids at the specific positions.

A heat map of the crotonylated site motifs illustrated the sequence patterns surrounding the Kcr. As shown in Figure 7, the amino acids showed similar characteristics of enrichment and depletion around the Kcr in the conidial and mycelial stages. In the human lung adenocarcinoma cell line H1299, alanine (A), aspartic acid (D), and glutamic acid (E) were significantly enriched in the region surrounding the Kcr (Weizhi Xu et al., 2017). In papaya (*Carica papaya* L.), lysine (K) and arginine (R) were enriched in the −10 to −5 and +5 to +10 regions around the Kcr (Liu et al., 2018). Our study showed consistent motifs in these species; it showed that A was enriched at the −2 to −1 positions, D and E were both enriched at the −2 to −1 and +1 to +4 positions, and K and R were both enriched at the −10 to −5 and +5 to +10 positions. In addition, some amino acids were depleted around Kcr. For instance, proline (P) and serine (S) were both depleted at −10 to −2 and +1 to +10 positions. These results suggest that the conserved amino acid sequence characteristics of crotonylation might be specifically identified by the enzyme catalyzing crotonylation.

### Conservation Analysis of Crotonylated Lysines in Other Species

Some studies have reported that modified lysines, including propionylated, acetylated, and malonylated lysines, are more conserved than unmodified lysines (Mo et al., 2015; Fan et al., 2017; Xu et al., 2019). To determine whether crotonylated lysines showed the same phenomenon, the crotonylated and uncrotonylated lysines in *T. rubrum* were compared to other species. The selected species included *Homo sapiens*, *Mus musculus*, *Escherichia coli* (strain_K12), and two fungal species (*C. albicans* and *Aspergillus flavus*). The results suggested that the crotonylated lysines were more conserved than uncrotonylated lysines in all these species (Supplementary Figure S7). The fact that whether PTMs are more likely to occur in conserved regions, or the modified regions, is less likely to change during evolution, deserves further exploration.

### Relations of Crotonylation and Protein Abundance

Crotonylation showed different abundances between the conidial and mycelial stages, and a large percentage of crotonylated proteins were specific to one stage. To investigate whether the specificity of crotonylated proteins to a particular stage was due to the greater protein abundance in that stage or whether crotonylation was likely to occur on more abundant proteins, we compared the crotonylome with the quantified whole-cell proteome studied previously (Xu et al., 2018). Among the 1190 proteins that were specifically crotonylated in conidia, 98 proteins were only expressed in conidia, and 146 proteins were common to the two stages but more abundant in conidia. These two categories of proteins only accounted for 20.5% of the 1190 conidia-specific crotonylated proteins. In addition, among the 380 proteins that were specifically crotonylated in mycelia, 32 proteins were only expressed in mycelia, and 155 proteins were more abundant in mycelia than in conidia. Proteins in these categories accounted for 49.2% of the 380 mycelia-specific crotonylated proteins. Based on these results, we conclude that the differences in crotonylation observed between the two stages were not due to differences in protein abundance.

### Crosstalk of Crotonylation With Acetylation, Succinylation, and Propionylation Based on KEGG, GO, and Domain Enrichment

**FIGURE 8:**
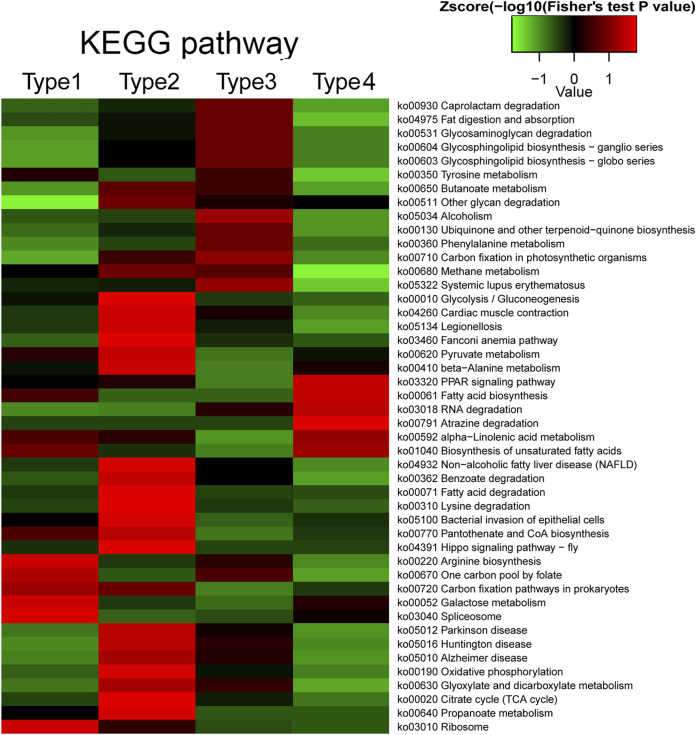
Crosstalk of crotonylation with acetylation, succinylation, and propionylation based on KEGG enrichment. Type 1 represents proteins commonly modified by crotonylation and acetylation; type 2 represents proteins commonly modified by crotonylation and succinylation; type 3 represents proteins commonly modified by crotonylation and propionylation; and type 4 represents proteins specifically modified by crotonylation.

Different PTMs can affect each other and work together to regulate protein function (Horita et al., 2018; Wu et al., 2019). We investigated the functions of proteins that are commonly modified by different kinds of PTM and the proteins specifically modified by crotonylation. The modified proteins were classified into four types, where type 1 represents proteins commonly modified by crotonylation and acetylation, type 2 represents proteins commonly modified by crotonylation and succinylation, type 3 represents proteins commonly modified by crotonylation and propionylation, and type 4 represents proteins specifically modified by crotonylation. In the KEGG pathway enrichment analysis, the four types of proteins displayed distinct enrichment patterns (Figure 8). Proteins commonly modified by crotonylation and acetylation (type 1) were significantly enriched in pathways including arginine biosynthesis, one carbon pool by folate, galactose metabolism, and ribosomes. Proteins commonly modified by crotonylation and succinylation (type 2) were mostly enriched in glycolysis/gluconeogenesis, pyruvate metabolism, benzoate degradation, fatty acid degradation, oxidative phosphorylation, and citrate cycle (TCA cycle). Proteins commonly modified by crotonylation and propionylation (type 3) were relatively enriched in pathways including caprolactam degradation, glycosaminoglycan degradation, alcoholism, and phenylalanine metabolism. Crotonylation-specific proteins (type 4) were mainly involved in pathways including the PPAR signaling pathway, fatty acid biosynthesis, RNA degradation, alpha-linolenic acid metabolism, and biosynthesis of unsaturated fatty acids. In the GO biological process, molecular function, cellular component, and protein domain enrichment analyses, all four types of protein showed obviously distinct enriched and depleted items (Supplementary Figure S8). All these results suggest that crotonylated proteins might participate individually or in collaboration with other PTMs in distinct regulatory pathways.

## Discussion

In our study, we identified 14,019 crotonylated sites on 3144 proteins in total, accounting for 30% of the *T. rubrum* proteome. Compared with the previously identified acetylation, propionylation, and succinylation, crotonylation and acetylation (5580 acetylated sites on 2422 proteins) are the two most abundant PTM types in *T. rubrum.* By contrast, propionylation and succinylation are rare in this species, modifying 115 and 284 proteins, respectively. Acetylation is widespread in a variety of organisms and is the most studied PTM type. Our comparisons showed a greater number of crotonylated proteins than acetylated proteins and a higher ratio of modified sites per protein for crotonylation than for acetylation (4.5 and 2.3, respectively). These results suggest that crotonylation is a highly abundant modification type in *T. rubrum*.

The distribution patterns of crotonylation in conidia and mycelia were different from those of the other three types of PTM. First, acetylation, succinylation, and propionylation were all more abundant in mycelia than in conidia; in particular, there were eight times more acetylated proteins in mycelia than in conidia. However, crotonylation was less abundant in mycelia than in conidia. Second, for the other three types of PTM, the most modified proteins were specific to either the conidial or the mycelial stage; the ratios of modified growth-stage-specific proteins to all modified proteins were 92% for acetylation, 92.2% for propionylation, and 77.8% for succinylation. However, growth-stage-specific crotonylated proteins represented only 49.9% of the total. More than half of crotonylated proteins were common to the conidial and mycelial stages.

Functional analysis showed that crotonylated proteins were mostly involved in various metabolic pathways, including the small molecule metabolic process, cellular amino acid metabolic process, and nucleotide metabolic process. In addition, translation-related processes were significantly enriched. Consistent with this, 92 ribosomal proteins were identified to be crotonylated. Ribosomes are the essential machines that catalyze protein synthesis. Ribosomal proteins are important constituents of each ribosome subunit and contribute to the assembly and folding of ribosomal subunits; thus, they have a function in translation (de la Cruz et al., 2015). In addition to their roles in protein synthesis, ribosomal proteins have been reported to participate in other regulation pathways. For instance, the ribosomal protein RPL14B has a specific role in developmental regulation, that is, it is critical for fertilization in *Arabidopsis* (Luo et al., 2020). Ribosomal proteins have also been shown to be related to carcinogenesis of nasopharyngeal carcinoma (NPC) (Sim et al., 2017). Four ribosomal proteins, uS8 (S8), uS4 (S9), eS31 (S27a), and uL14 (L23), were found to be differentially expressed in NPC cell lines compared with a nonmalignant nasopharyngeal epithelial cell line (NP69) (Sim et al., 2017). In our study, although crotonylation was more abundant in conidia than mycelia overall, the crotonylated sites on ribosomal proteins were more abundant in mycelia than in conidia. Mycelia represent a vigorous growth state in which protein synthesis is significantly activated (Xu et al., 2018). Further investigation is required to determine the significant roles of the abundant crotonylation on ribosomal proteins and whether the more abundant crotonylation modification on ribosomal proteins in mycelia is related to increased protein synthesis. Furthermore, some proteins related to virulence were crotonylated, including secreted proteases and peptidases, as well as some efflux transporters. Due to the essential impact of reversible PTM on protein function and activity, these results suggested that crotonylation may also have a role in fungal pathogenicity (Scott, 2012; Cain et al., 2014). However, the specific regulatory mechanism requires further exploration.

When compared between the conidial and mycelial stages, the crotonylated proteins showed a similar function distribution based on GO classification, suggesting the overall functional similarity between the two stages. However, the crotonylated proteins showed some differences in function enrichment analysis. These results suggested that crotonylated proteins were involved in some distinct pathways in different growth stages of this fungus. Based on the GO enrichment of the biological process, the nucleotide biosynthetic and metabolic-related processes were significantly enriched in the conidial stage, including the nucleoside phosphate metabolic process, nucleotide metabolic process, nucleoside phosphate biosynthetic process, and nucleotide biosynthetic process. Conidia are the dormant stage of *T. rubrum*. In our previous study, it has been suggested that a pool of mRNAs were pre-stored in conidia (Xu et al., 2011). In the existence of nutrients, these mRNAs could be activated rapidly to initiate translation and promote conidia germination, thus facilitating infection (Xu et al., 2011). The fact that whether these crotonylated proteins are involved in the synthesis and metabolism of these pre-stored mRNAs deserves further investigation.

Histone PTMs have essential roles in gene expression regulation (Bennett et al., 2019). For instance, acetylations reduce the positive charge of histones and attenuate their interactions with the negatively charged DNA backbone, thereby facilitating gene transcription (Bennett et al., 2019). In contrast, hypoacetylation is associated with transcriptional suppression (Lee et al., 1993; Bennett et al., 2019). Similarly, histone crotonylation is involved in transcription activation (Gowans et al., 2019). Studies have showed that histone crotonylation catalyzed by acetyltransferase p300 stimulates transcription to a greater degree than histone acetylation (Sabari et al., 2015). Various modifications on histones comprise a “histone code,” enabling enzymatic PTM “readers” to recognize specific PTMs and “writers” and “erasers” to add or remove modifications, respectively (Strahl and Allis, 2000). Furthermore, the combinations of different types of PTMs on histones synergistically fulfill specific functions, which could be different from or go beyond the roles of the individual mark (Strahl and Allis, 2000). In our study, crotonylation, acetylation, succinylation, and propionylation were all found on histones. A total of 53 crotonylated sites were identified on histones, and 32 of these crotonylated sites were commonly modified by at least one of the other three types of PTM. One significant modified site was H3K56, whose acetylation (H3K56ac) has been the object of many studies. In fission yeast, H3K56ac was found to be the bona fide mark for transcription-dependent histone turnover (Yang et al., 2016). In human embryonic stem cells, H3K56ac was shown to be involved in the core transcriptional network of pluripotency (Xie et al., 2009). In addition, H3K56ac was identified as an essential factor for the small RNA biogenesis pathway through its role in homologous recombination (Zhang et al., 2014). In our study, H3K56 was commonly modified by acetylation, succinylation, propionylation, and crotonylation. All these PTMs may coordinate with each other to regulate this histone site. In addition to the commonly modified sites, different PTMs showed differential abundances on histones and displayed distinct modification patterns in conidia and mycelia. Crotonylation was the most abundant modification type on histones, and propionylation was the least abundant among the four types of PTM. When compared between the conidial and mycelial stages, the number of propionylated sites on histones was similar in the two stages; however, succinylated and acetylated sites on histones were both more abundant in mycelia than in conidia. By contrast, crotonylated sites on histones were more abundant in conidia than in mycelia. Thus, we speculate that each type of histone PTM may have distinct roles but that they may also cooperate with each other in epigenetic regulation.

We also analyzed the amino acid sequence patterns around the Kcr, which may reflect the specific recognition of enzymes that catalyze crotonylation. Based on comparisons with previous studies, the amino acid sequence patterns surrounding crotonylated sites are different from those around acetylated, succinylated, and propionylated sites. In addition, the amino acids surrounding the Kcr show similar characteristics between the conidial and mycelial stages. This is in contrast to acetylation, propionylation, and succinylation, which all display different surrounding amino acid patterns between conidia and mycelia. These results further reflect the different characteristics of crotonylation compared with the other three types of PTM. Lysine acetylation is dynamically regulated by two groups of enzymes: lysine acetyltransferases (writers) that add acetyl groups to lysine and deacetylases (erasers) that remove these modifications (Hyndman and Knepper, 2017). Other non-acetylated lysine acylations are also catalyzed by these two groups of enzymes, which have been shown to have an expanded repertoire of acyltransferase or deacylase activities (Sabari et al., 2017). For instance, p300 is considered a promiscuous acyltransferase that could catalyze lysine propionylation (Chen et al., 2007), butyrylation (Chen et al., 2007), crotonylation (Sabari et al., 2015), *β*-hydroxybutyrylation (Kaczmarska et al., 2017), succinylation (Sabari et al., 2017), and glutarylation (Tan et al., 2014). The deacetylase SIRT5 has robust demalonylase, desuccinylase, and deglutarylase activities but very weak deacetylase activity (Du et al., 2011; Peng et al., 2011; Park et al., 2013; Tan et al., 2014). However, fungal acetyltransferases and deacetylases show low homology to their well-studied counterparts in animals and plants, and little is known about the enzymes that catalyze non-acetylated lysine acylation in fungi. The different patterns of amino acids surrounding the different types of modified lysine might indicate that the enzymes catalyzing each type of PTM recognize distinct substrate sequences.

Fungal acetyltransferases and deacetylases are also modified by various PTMs. In our study, 11 lysine acetyltransferases and five deacetylases were found to be crotonylated; eight of these enzymes were commonly modified by acetylation (Supplementary Table S9). These results suggest that crotonylation and acetylation might be involved in the regulation of PTMs. In addition to controlling the balance of PTMs in specific states, acetyltransferases and deacetylases play vital roles in fungal development. Our previous study showed that inhibition of acetyltransferases or deacetylases inhibited the fungal growth and induced cell apoptosis (Xu et al., 2018). Focus on these two groups of enzymes and their PTMs would provide new insights to guide studies of antifungal agents.

In our study, crosstalk of crotonylation with each of the other three types of PTMs showed distinct regulatory roles. As well as the fact that PTMs were already identified in *T. rubrum*, many other types of PTMs were also found to exist in this fungus, including butyrylation, dimethylation, glutarylation, ubiquitination, and malonylation (Xu et al., 2019). Different PTMs display distinct functions and characteristics, but they may collaborate with others to participate in biological regulation. All these types of PTMs constitute a complex network that executes their regulatory roles.

Overall, our study is the first large-scale identification of crotonylation in dermatophytes and human pathogenic filamentous fungi. These results represent a foundation for further research on the PTM regulation mechanism in these fungi, which could facilitate the development of improved therapeutic approaches against these medically significant fungi.

## Data Availability

The datasets presented in this study can be found in online repositories. The names of the repository/repositories and accession number(s) can be found below: http://www.peptideatlas.org/, PASS01413.
