# Supplementary material for: Proteome-Wide Identification and Functional Analysis of Lysine Crotonylation in Trichophyton rubrum Conidial and Mycelial Stages
Source: Front Genet. 2022 Mar 10;13:832668. doi: 10.3389/fgene.2022.832668 (PMC8960058; doi:10.3389/fgene.2022.832668)
Supplement: Supplementary file 5 [file DataSheet1.PDF]

Supplementary Figures

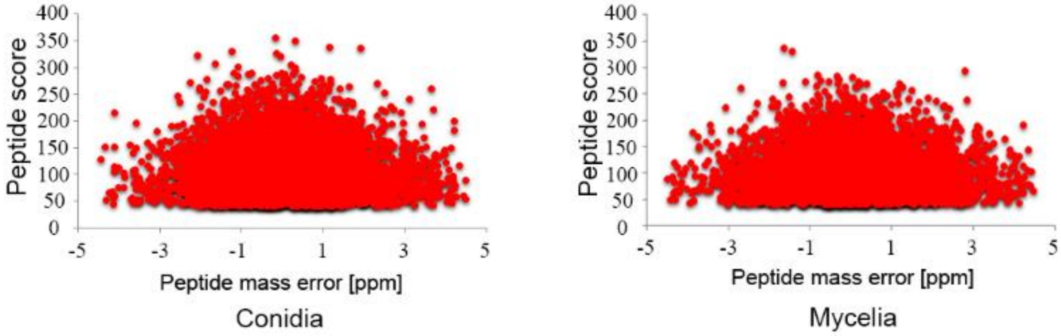

Figure S1. The mass errors and scores for crotonylated peptide identification in each conidial and mycelial stage respectively.

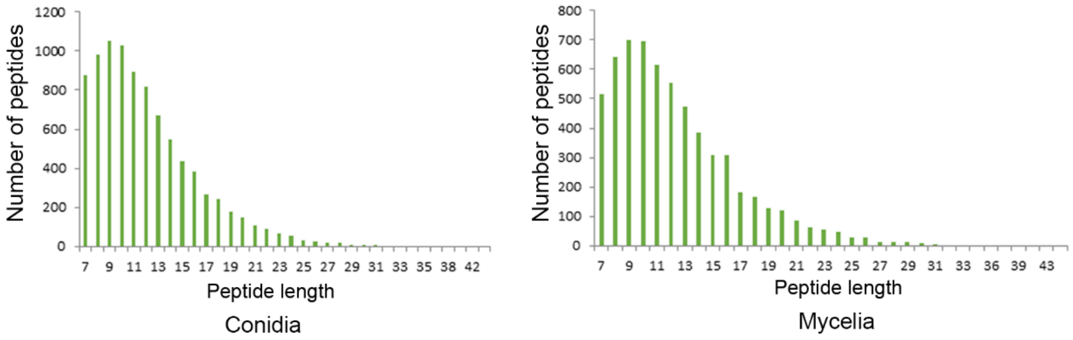

Figure S2. The peptide length distribution in each conidial and mycelial stage respectively.

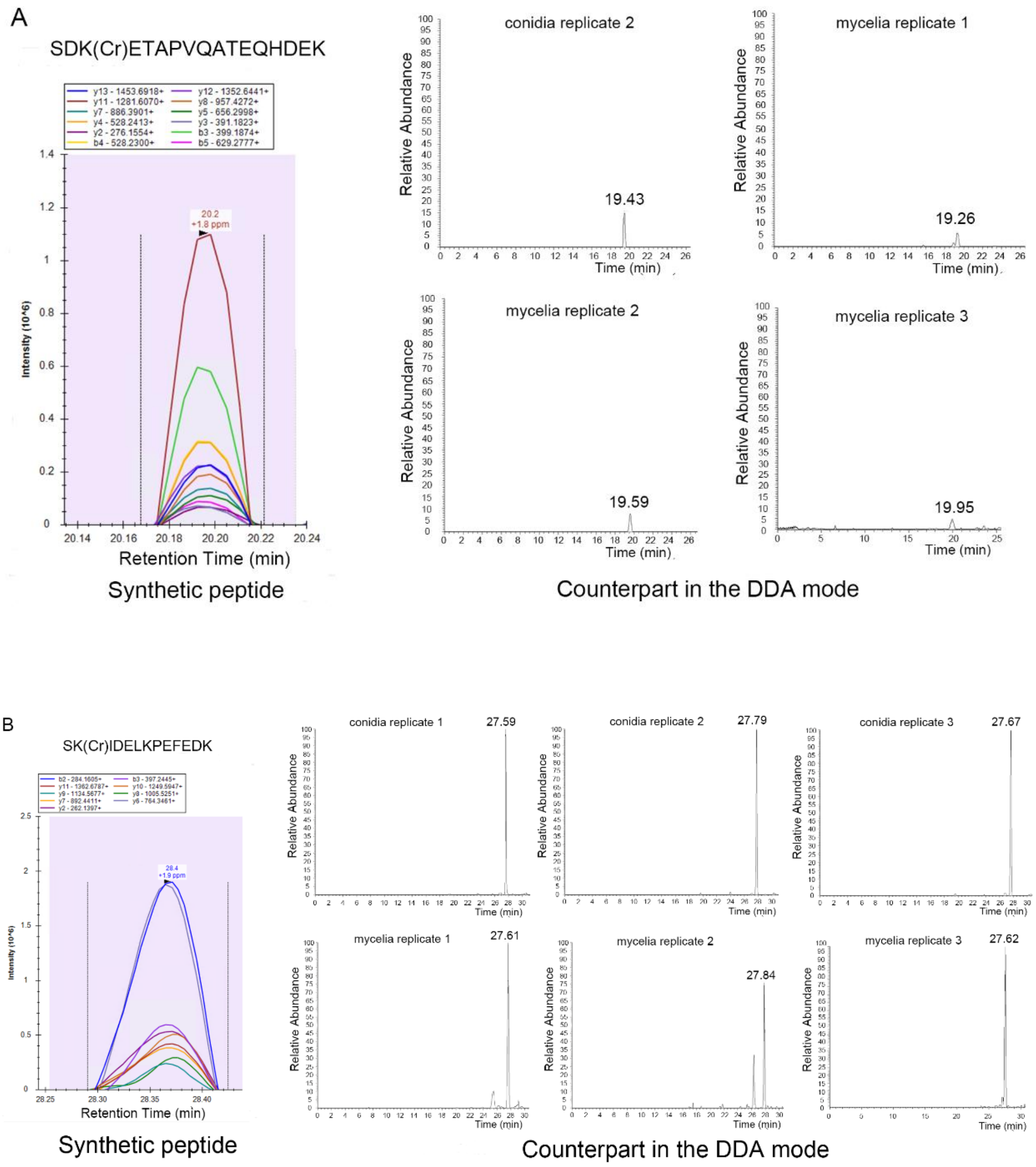

Figure S3. The retention time of the synthetic peptides and their counterparts in the LC-MS/MS identification. (A) The retention time for peptide SDK(Cr)ETAPVQATEQHDEK. (B) The retention time for peptide SK(Cr)IDELKPEFEDK.

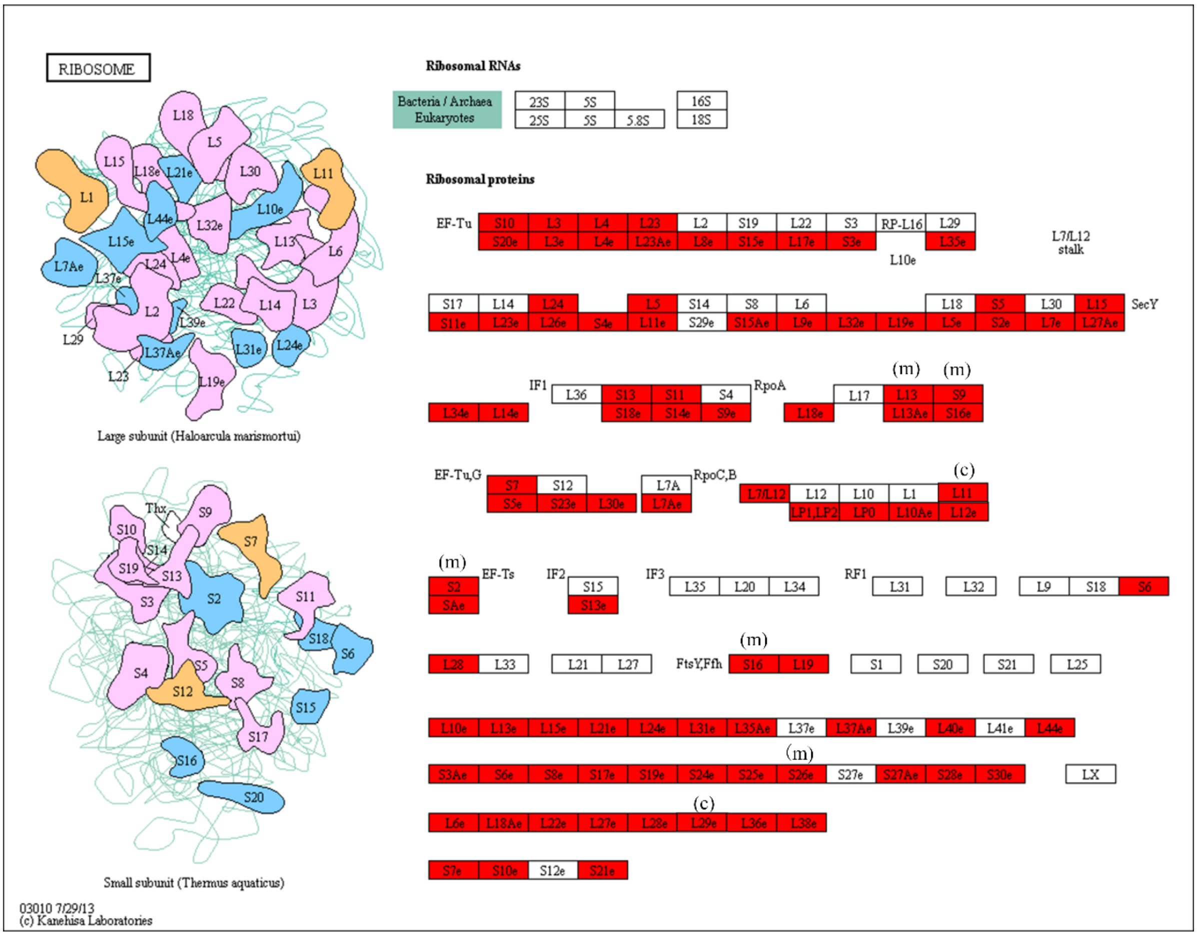

Figure S4. Ribosomal proteins modified by crotonylation. The ribosomal proteins with red color indicate that they are modified with crotonylation. The letter “c” in the bracket indicates that the protein is only modified in conidial stage; the letter “m” in the bracket indicates that the protein is only modified in mycelial stage.

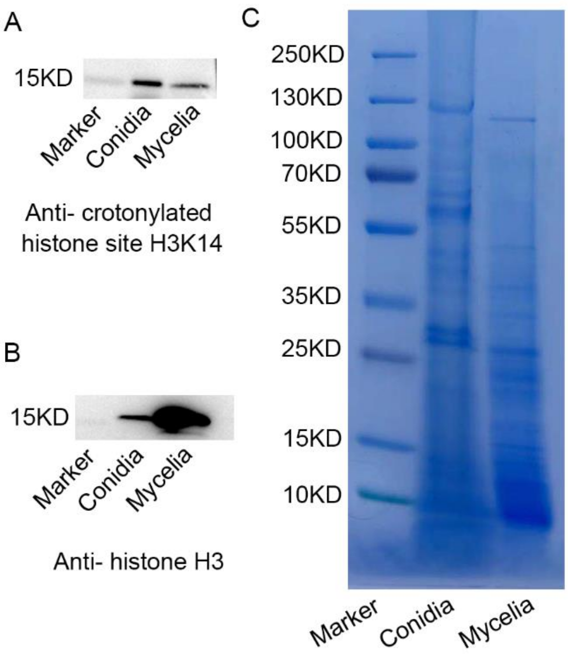

Figure S5. Western blot validating the crotonylated site on histone. (A) Histone site H3K14 crotonylation was validated using a site specific antibody, anti- crotonylated histone H3K14. (B) Western blot of histone H3. (C) Loading control in western blot analysis. Equal amount (20 μg) of whole-cell protein sample was loaded for each conidial and mycelial stage.

A

Logo	Motif	Motif Score	Foreground		Background		Fold Increase
			Matches	Size	Matches	Size	
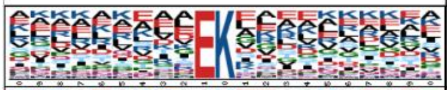	.........EK..........	16	1504	10571	14306	181000	1.8
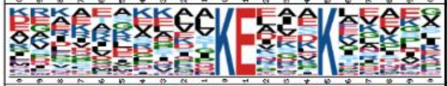	.........KE...K.....	26.32	179	9067	1029	166694	3.2
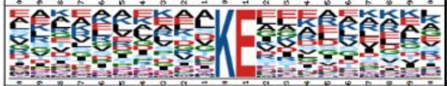	..........KE.........	16	1088	8888	10965	165665	1.85
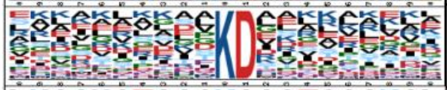	..........KD.........	16	974	7800	9662	154700	2
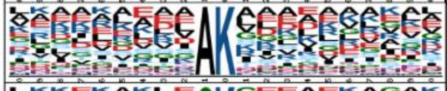	.........AK..........	16	799	6826	12142	145038	1.4
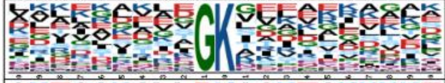	.........GK..........	16	714	6027	11159	132896	1.41
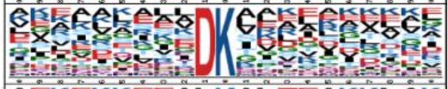	.........DK..........	16	637	5313	7767	121737	1.88
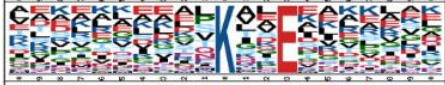	..........K..E.......	16	537	4676	8404	113970	1.56
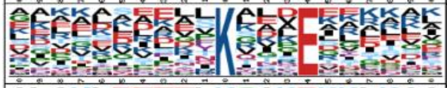	..........K...E......	16	468	4139	8012	105566	1.49
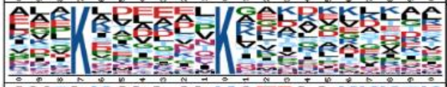	...K.......K..........	16	393	3671	6284	97554	1.66
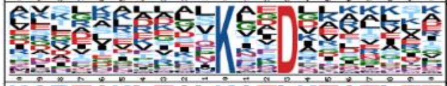	..........K..D.......	16	359	3278	5911	91270	1.69
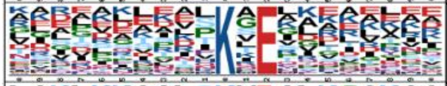	..........K.E........	16	272	2919	4657	85359	1.71
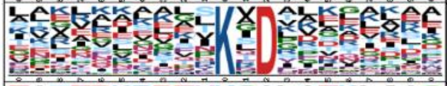	..........K.D........	16	257	2647	4225	80702	1.85
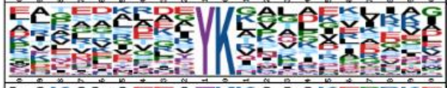	.........YK..........	16	171	2390	2526	76477	2.17
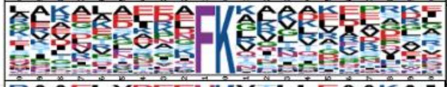	.........FK..........	16	191	2219	3258	73951	1.95
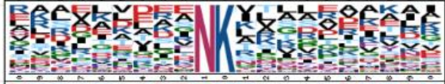	.........NK..........	16	204	2028	3723	70693	1.91
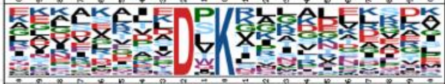	........D.K..........	15.65	180	1824	3436	66970	1.92
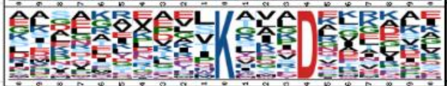	..........K...D......	10.22	176	1644	4093	63534	1.66
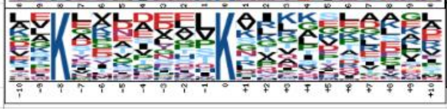	..K........K..........	10.75	155	1468	3577	59441	1.75

B

Logo	Motif	Motif Score	Foreground		Background		Fold Increase
			Matches	Size	Matches	Size	
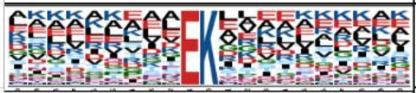	..........EK..........	16	1090	7641	14306	181000	1.8
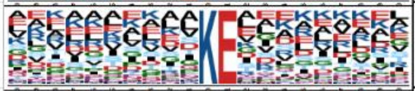	..........KE.........	16	885	6551	11994	166694	1.88
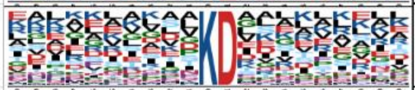	..........KD.........	16	694	5666	9662	154700	1.96
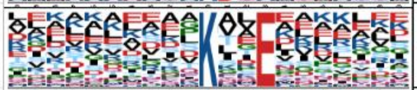	..........K..E........	16	556	4972	10653	145038	1.52
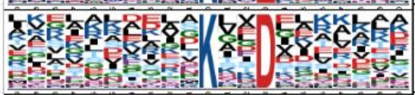	..........K..D........	16	456	4416	8557	134385	1.62
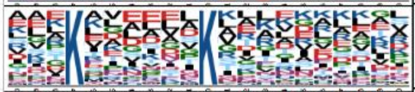	...K......K..........	16	404	3960	8195	125828	1.57
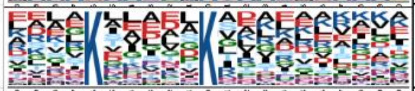	....K.....K..........	16	350	3556	7047	117633	1.64
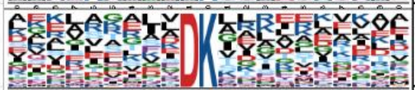	.........DK..........	16	318	3206	5930	110586	1.85
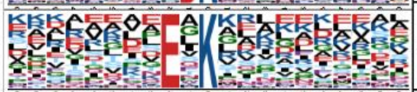	........E.K..........	13.92	283	2888	6394	104656	1.6
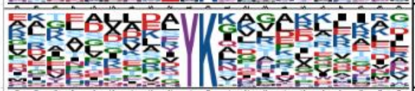	.........YK..........	13.13	141	2605	2685	98262	1.98
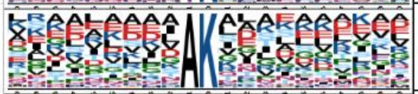	.........AK..........	13.08	334	2464	8606	95577	1.51
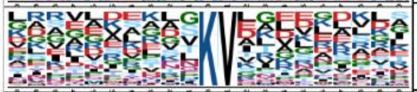	..........KV.........	10.61	210	2130	5342	86971	1.61
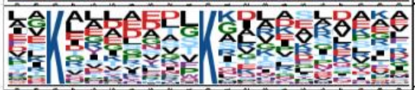	..K.......K..........	11.08	189	1920	4803	81629	1.67
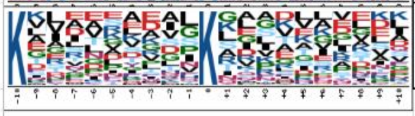	K.........K..........	9.54	160	1731	4237	76826	1.68

Figure S6. The significantly enriched and conserved crotonylated-site motif in the (A) conidial and (B) mycelial stages.

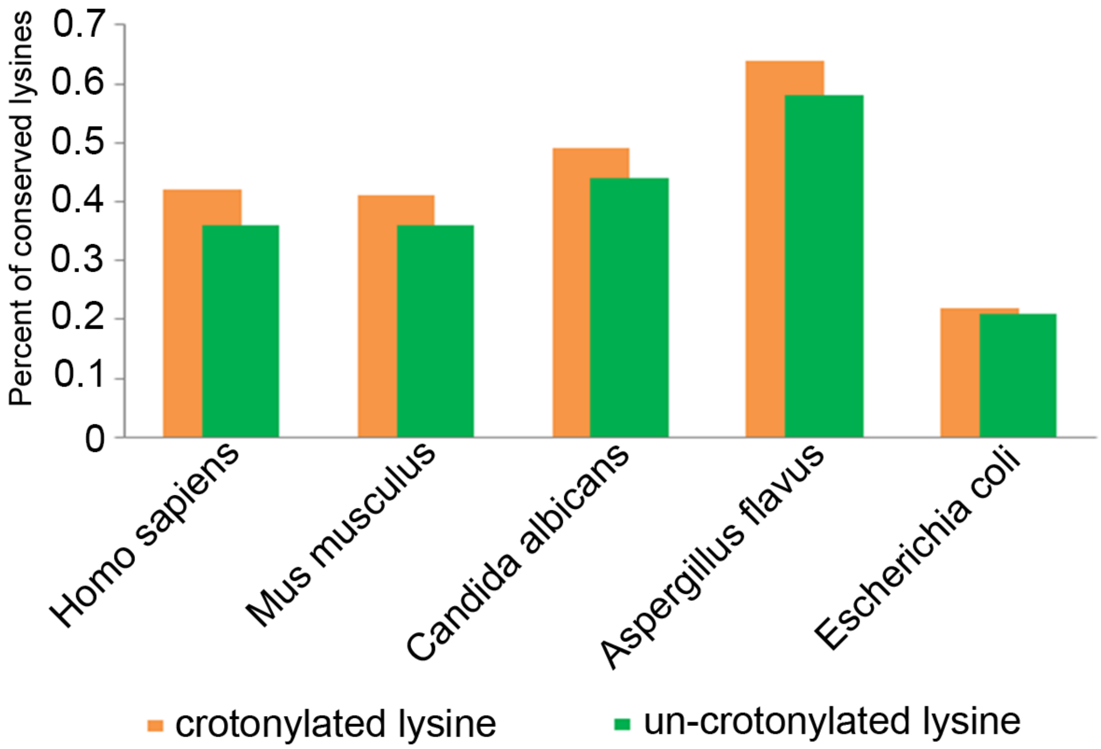

Figure S7. Conservation analysis of crotonylated lysine and un-crotonylated lysine in different species.

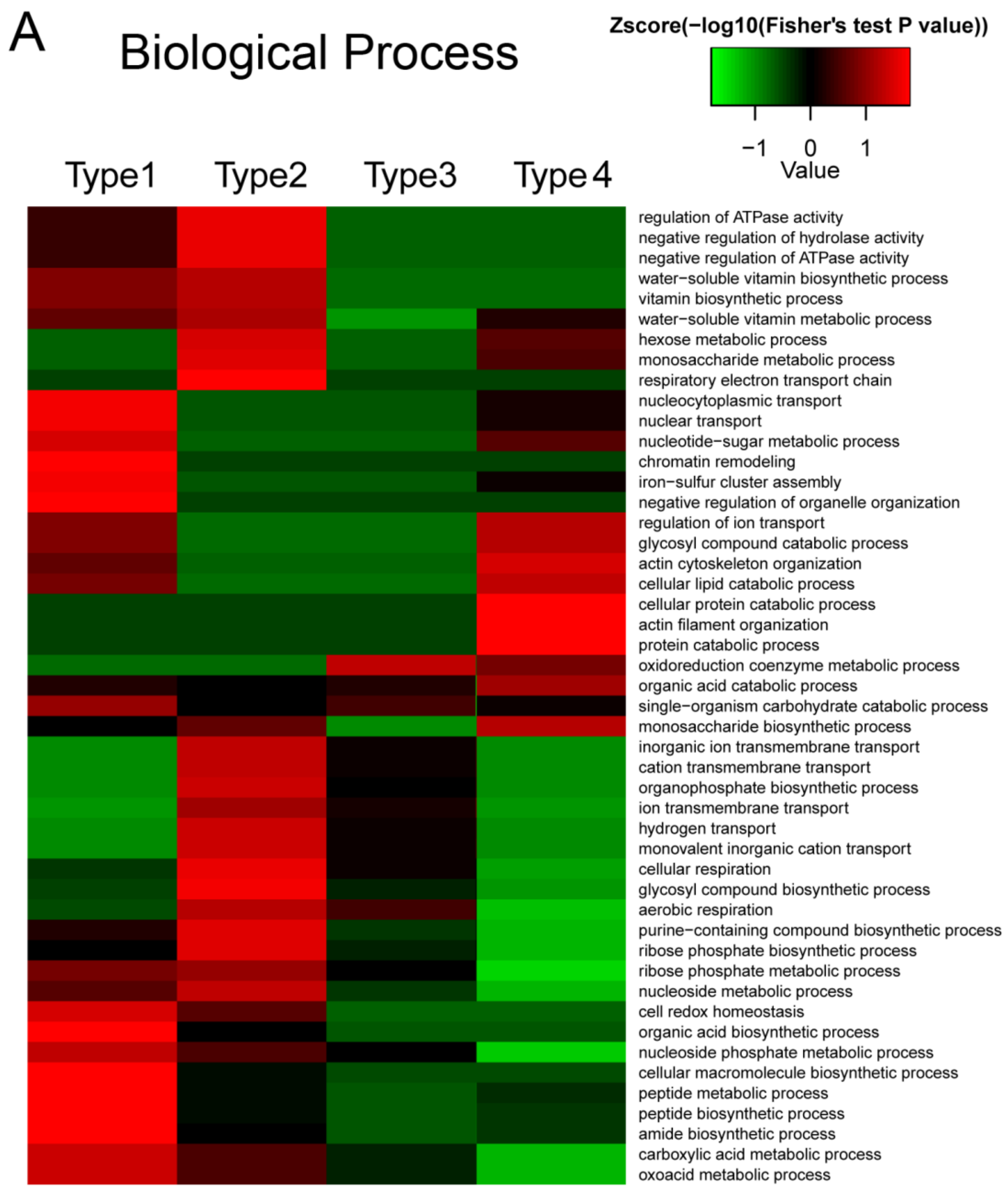
A
Biological Process
Zscore(−log10(Fisher's test P value))
−1
0
1
Value
Type1
Type2
Type3
Type4
regulation of ATPase activity
negative regulation of hydrolase activity
negative regulation of ATPase activity
water−soluble vitamin biosynthetic process
vitamin biosynthetic process
water−soluble vitamin metabolic process
hexose metabolic process
monosaccharide metabolic process
respiratory electron transport chain
nucleocytoplasmic transport
nuclear transport
nucleotide−sugar metabolic process
chromatin remodeling
iron−sulfur cluster assembly
negative regulation of organelle organization
regulation of ion transport
glycosyl compound catabolic process
actin cytoskeleton organization
cellular lipid catabolic process
cellular protein catabolic process
actin filament organization
protein catabolic process
oxidoreduction coenzyme metabolic process
organic acid catabolic process
single−organism carbohydrate catabolic process
monosaccharide biosynthetic process
inorganic ion transmembrane transport
cation transmembrane transport
organophosphate biosynthetic process
ion transmembrane transport
hydrogen transport
monovalent inorganic cation transport
cellular respiration
glycosyl compound biosynthetic process
aerobic respiration
purine−containing compound biosynthetic process
ribose phosphate biosynthetic process
ribose phosphate metabolic process
nucleoside metabolic process
cell redox homeostasis
organic acid biosynthetic process
nucleoside phosphate metabolic process
cellular macromolecule biosynthetic process
peptide metabolic process
peptide biosynthetic process
amide biosynthetic process
carboxylic acid metabolic process
oxoacid metabolic process

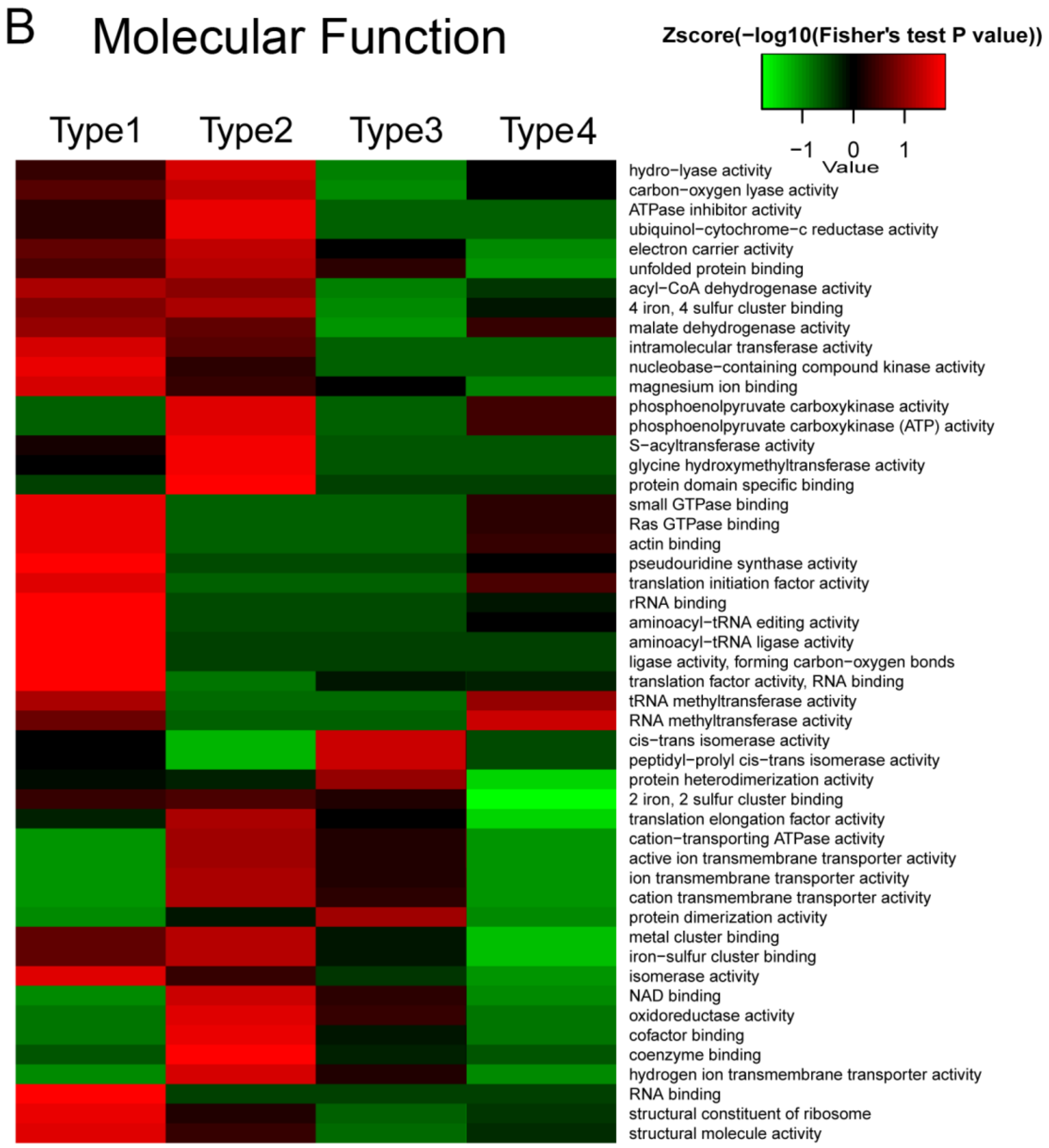

B
Molecular Function
Zscore(−log10(Fisher's test P value))
−1
0
1
Value
Type1
Type2
Type3
Type4
hydro−lyase activity
carbon−oxygen lyase activity
ATPase inhibitor activity
ubiquinol−cytochrome−c reductase activity
electron carrier activity
unfolded protein binding
acyl−CoA dehydrogenase activity
4 iron, 4 sulfur cluster binding
malate dehydrogenase activity
intramolecular transferase activity
nucleobase−containing compound kinase activity
magnesium ion binding
phosphoenolpyruvate carboxykinase activity
phosphoenolpyruvate carboxykinase (ATP) activity
S−acyltransferase activity
glycine hydroxymethyltransferase activity
protein domain specific binding
small GTPase binding
Ras GTPase binding
actin binding
pseudouridine synthase activity
translation initiation factor activity
rRNA binding
aminoacyl−tRNA editing activity
aminoacyl−tRNA ligase activity
ligase activity, forming carbon−oxygen bonds
translation factor activity, RNA binding
tRNA methyltransferase activity
RNA methyltransferase activity
cis−trans isomerase activity
peptidyl−prolyl cis−trans isomerase activity
protein heterodimerization activity
2 iron, 2 sulfur cluster binding
translation elongation factor activity
cation−transporting ATPase activity
active ion transmembrane transporter activity
ion transmembrane transporter activity
cation transmembrane transporter activity
protein dimerization activity
metal cluster binding
iron−sulfur cluster binding
isomerase activity
NAD binding
oxidoreductase activity
cofactor binding
coenzyme binding
hydrogen ion transmembrane transporter activity
RNA binding
structural constituent of ribosome
structural molecule activity

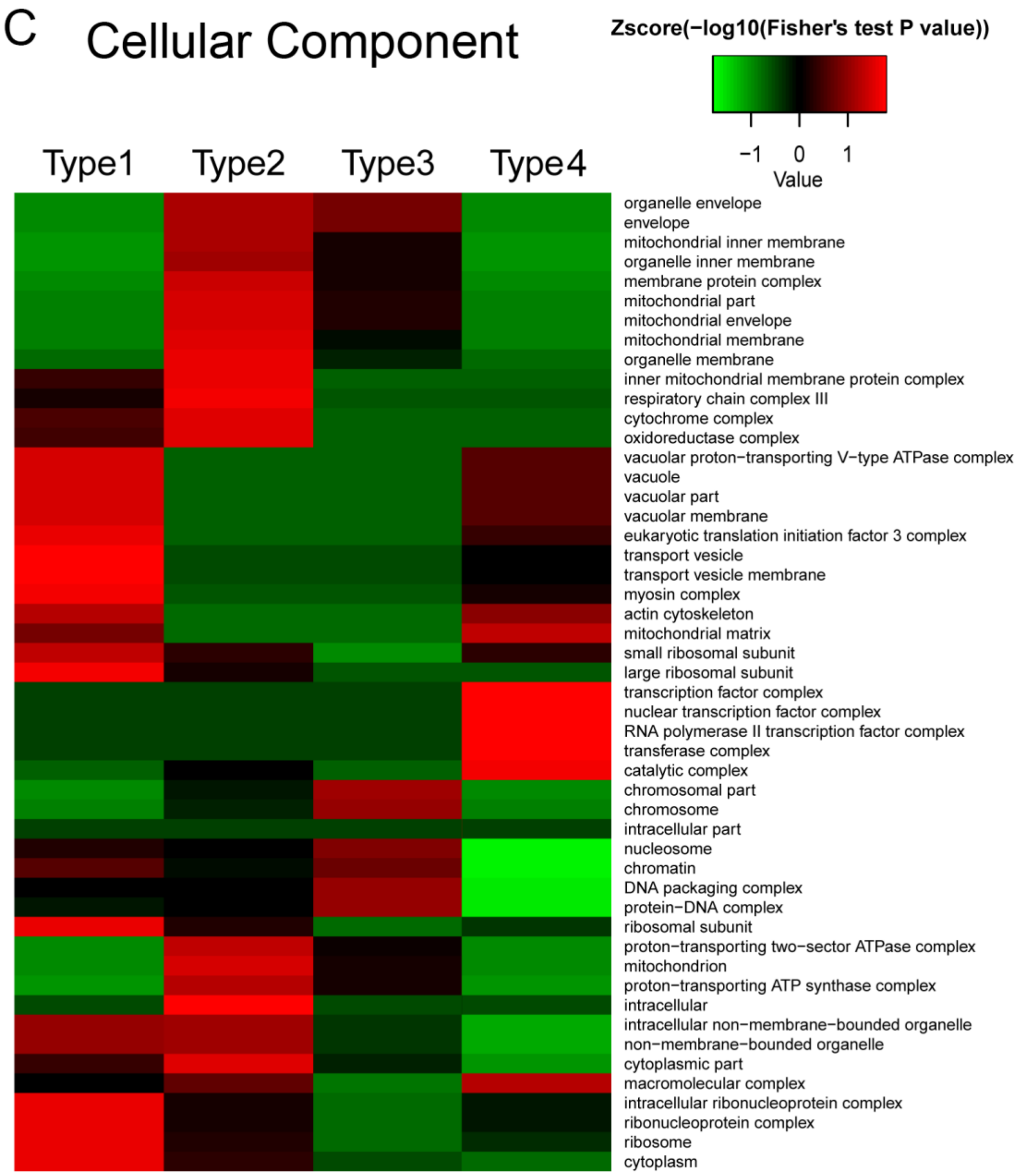
C
Cellular Component
Zscore(−log10(Fisher's test P value))
−1
0
1
Value
Type1
Type2
Type3
Type4
organelle envelope
envelope
mitochondrial inner membrane
organelle inner membrane
membrane protein complex
mitochondrial part
mitochondrial envelope
mitochondrial membrane
organelle membrane
inner mitochondrial membrane protein complex
respiratory chain complex III
cytochrome complex
oxidoreductase complex
vacuolar proton−transporting V−type ATPase complex
vacuole
vacuolar part
vacuolar membrane
eukaryotic translation initiation factor 3 complex
transport vesicle
transport vesicle membrane
myosin complex
actin cytoskeleton
mitochondrial matrix
small ribosomal subunit
large ribosomal subunit
transcription factor complex
nuclear transcription factor complex
RNA polymerase II transcription factor complex
transferase complex
catalytic complex
chromosomal part
chromosome
intracellular part
nucleosome
chromatin
DNA packaging complex
protein−DNA complex
ribosomal subunit
proton−transporting two−sector ATPase complex
mitochondrion
proton−transporting ATP synthase complex
intracellular
intracellular non−membrane−bounded organelle
non−membrane−bounded organelle
cytoplasmic part
macromolecular complex
intracellular ribonucleoprotein complex
ribonucleoprotein complex
ribosome
cytoplasm

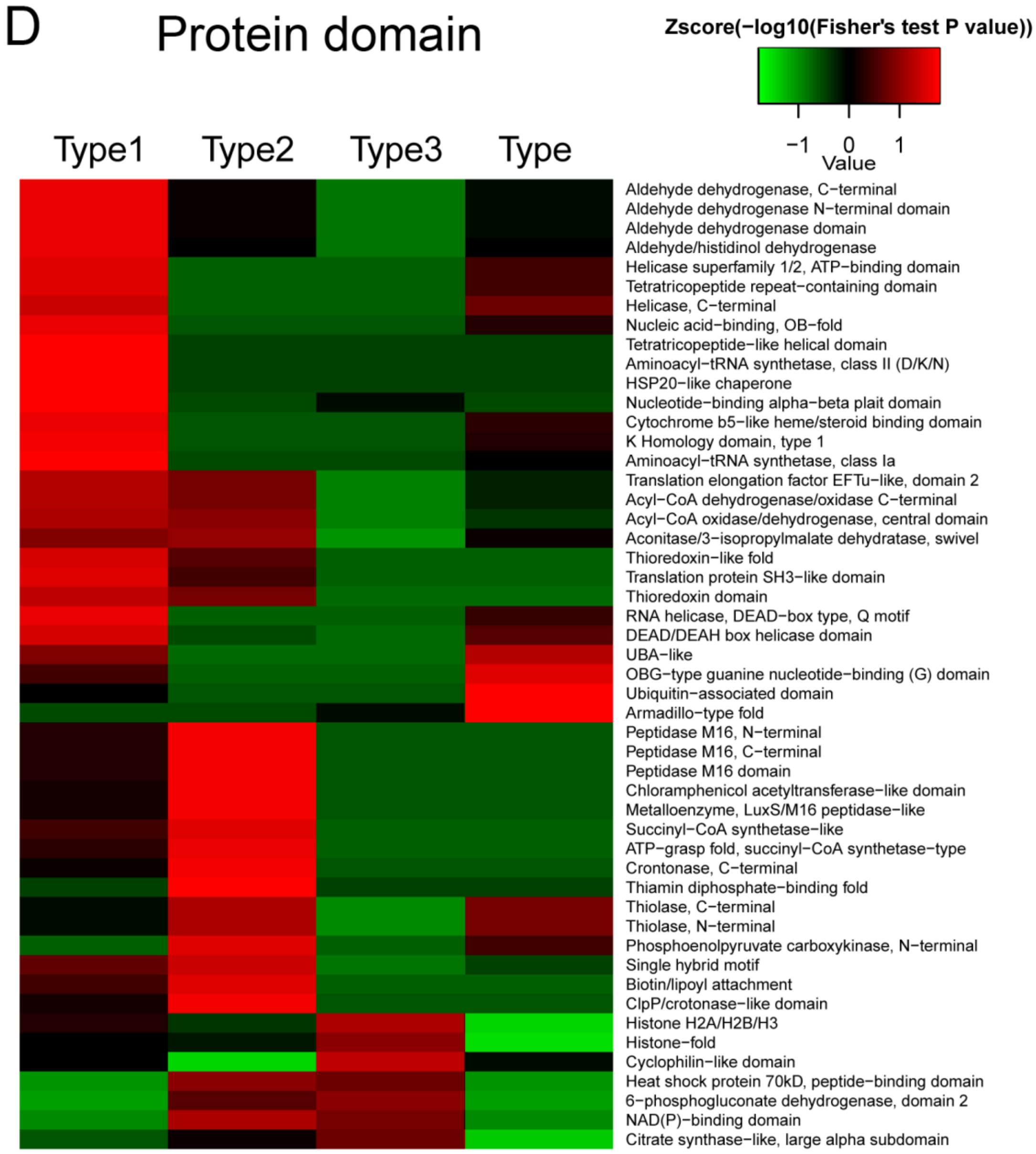

Figure S8. Crosstalk of crotonylated proteins with acetylated, succinylated and propionylated proteins. Type 1: proteins commonly modified by crotonylation and acetylation; Type 2: proteins commonly modified by crotonylation and succinylation; Type 3: proteins commonly modified by crotonylation and propionylation; Type 4: proteins specific modified by crotonylation. GO enrichment of the four types of proteins based on (A) biological process, (B) molecular function and (C) cellular component. (D) Enrichment of proteins based on protein domain.
